# Comparative effectiveness of noninvasive therapeutic interventions for myofascial pain syndrome: a network meta-analysis of randomized controlled trials

**DOI:** 10.1097/JS9.0000000000000860

**Published:** 2023-11-07

**Authors:** Chang Liu, Yang Wang, Wenli Yu, Junai Xiang, Guoyong Ding, Weihua Liu

**Affiliations:** a School of Nursing; b School of Public Health, Shandong First Medical University and Shandong Academy of Medical Sciences, Jinan, Shandong; c Department of Anesthesiology, Tianjin First Center Hospital, Tianjin, People’s Republic of China; d Department of Plastic and Reconstructive Surgery, Cell and Matrix Research Institute, Kyungpook National University School of Medicine, Daegu, Korea

**Keywords:** myofascial pain, network meta-analysis, noninvasive methods, systematic review

## Abstract

**Background:**

Myofascial pain syndrome (MPS) has an impact on physical health and quality of life for patients, with various noninvasive methods used for relieving myofascial pain. The authors aimed to compare the effectiveness of different noninvasive therapeutic interventions for MPS.

**Materials and methods:**

The authors searched PubMed, Embase, CINAHL Complete, Web of Science, Cochrane, and Scopus to identify randomized controlled trials describing the effects of any noninvasive treatments in patients with MPS. The primary outcome was pain intensity, while pressure pain threshold and pain-related disability were secondary outcomes.

**Results:**

The analysis included 40 studies. Manual therapy [mean difference (MD) of pain: −1.60, 95% CI: −2.17 to −1.03; MD of pressure pain threshold: 0.52, 95% CI: 0.19 to 0.86; MD of pain-related disability: −5.34, 95% CI: −8.09 to −2.58], laser therapy (MD of pain: −1.15, 95% CI: −1.83 to −0.46; MD of pressure pain threshold: 1.00, 95% CI: 0.46 to 1.54; MD of pain-related disability: −4.58, 95% CI: −7.80 to −1.36), extracorporeal shock wave therapy (MD of pain: −1.61, 95% CI: −2.43 to −0.78; MD of pressure pain threshold: 0.84, 95% CI: 0.33 to 1.35; MD of pain-related disability: −5.78, 95% CI: −9.45 to −2.12), and ultrasound therapy (MD of pain: −1.54, 95% CI: −2.24 to −0.84; MD of pressure pain threshold: 0.77, 95% CI: 0.31 to 1.22) were more effective than no treatment.

**Conclusion:**

Our findings support that manual therapy, laser therapy, and extracorporeal shock wave therapy could effectively reduce pain intensity, pressure pain threshold, and pain-related disability with statistical significance when compared with placebo. This finding may provide clinicians with appropriate therapeutic modalities for patients with MPS among different scenarios.

## Introduction

Highlights

- Different noninvasive methods were used for easing myofascial pain syndrome.
- We used network meta-analysis to assess the effects of different noninvasive methods on pain intensity, pressure pain threshold, and disability.
- Manual therapy, laser therapy, and extracorporeal shock wave therapy demonstrated effectiveness in alleviating pain, increasing the pressure pain threshold, and decreasing pain-related disability.

Myofascial pain syndrome (MPS), originating from myofascial trigger points (MTrPs), is a common chronic musculoskeletal pain syndrome, with 30% to 85% incidence rate in patients with musculoskeletal pain^1^. MPS is a leading cause of chronic and persistent regional pain, which includes pain, local tenderness, limited joint movement, and other symptoms^1^. The incidence of MPS is more commonly in females; however, its etiology is controversial and is not yet fully understood^1,2^. The disease affects a wide range of people, and has also become a common phenomenon among college students, showing a trend in younger patients^3^. In the absence of timely treatment, MPS-associated pain symptoms can lead to dysfunction, disability and economic loss^4,5^. Moreover, MPS is detrimental to physical health and reduces the quality of life, making patients suffer from unpleasant experiences. In addition, it poses a certain threat to mental health and raises the level of anxiety and depression^3,6,7^.

With the development of medical technology, various treatments for MPS have emerged^8^. More specifically, the treatments of MPS involve in invasive and noninvasive methods^9,10^. Drug injection therapy and dry needle treatment are listed as invasive methods, whereas noninvasive includes manual therapy, hot package, extracorporeal shock wave therapy, ultrasound, exercise, transcutaneous electrical stimulation, and medicine recommended for the treatment of MTrPs^2,9,11–13^. In studies comparing invasive and noninvasive methods, the same effect for pain relief is observed and the side effects of noninvasive MPS therapy are less impactful^14–17^. Therefore, it is indispensable to examine the effectiveness of intervention with different noninvasive therapies in patients with myofascial pain. At present, there are several meta-analyses that explore the effectiveness of different noninvasive therapies on pain intensity and related indicators^17–23^. One systematic review concluded that the effect of manual therapy on pain intensity and pressure pain threshold was mixed, with a moderate effect size, and a moderate to high risk of bias in the included studies^24^. Nevertheless, other systematic reviews suggest that physical exercise programs and laser therapy may be effective methods to manage pain intensity and pressure pain threshold in MTrPs patients^18,22^. Although previous randomized controlled trials (RCTs), systematic reviews, and meta-analyses compared two types of treatments, the evidence that comparing the effectiveness of all types of noninvasive methods appears insufficient. Furthermore, it is not possible to investigate the effectiveness of these noninvasive therapies in standard pairwise meta-analyses.

Network meta-analysis (NMA) can estimate the effectiveness of intervention by using evidence of direct and indirect comparison^25^. By using NMA, a comparison of the effectiveness on pain of more than two types of noninvasive therapy is performed. Although there were evidences that several different noninvasive interventions were effective in pain relief compared with the control treatment^12,26,27^, it is difficult to comprehensively compare the relative effectiveness of different noninvasive modalities due to their wide variety. As such, we aimed to investigate the relative effectiveness of different noninvasive treatments in a NMA, that interprets the entire body of evidence, even if a direct comparison of the two types of intervention does not exist^28^.

## Methods

This NMA was fully reported according to the PRISMA (Preferred Reporting Items for Systematic Reviews and Meta-Analyses) statement, and AMSTAR 2 (Assessing the methodological quality of systematic reviews) guidelines^29,30^. This study protocol was prospectively registered on PROSPERO (CRD42023400783, https://www.crd.york.ac.uk/prospero/display_record.php?ID=CRDXXX).

### Information sources

We searched six electronic databases from their inception through 31 May 2023: PubMed, Embase, CINAHL Complete, Web of Science, Cochrane, and Scopus.

### Search strategy

A search strategy was developed and implemented under the guidance of experts on library services from one university, which included three terms for (1) treatment, (2) myofascial pain syndrome, and (3) randomized controlled trials. According to different databases, this strategy was adjusted to meet their requirements. The detailed search strategy of the six databases is listed in Supplemental Material 1 (Supplemental Digital Content 1, http://links.lww.com/JS9/B312). In addition, the reference lists of studies included in this review, and the reference lists studies included in previous reviews were manually screened to identify additional studies.

### Eligibility criteria and study selection

A study must have met the following criteria to be included: (1) RCTs; (2) participants (aged >18) were diagnosed with MPS; (3) participants underwent different noninvasive treatments in different groups; (4) provision of available outcome data; and (5) the included studies were limited to those published in English. We excluded the studies that noninvasive and invasive treatments existed simultaneously.

We also included the following comparators: (1) primary complaint was myofascial pain; (2) any type of noninvasive therapy which was different from the intervention group or no treatment, sham treatment, placebo treatment, and control treatment; and (3) reported outcomes including pain intensity, pressure pain threshold, or pain-related disability. If the control group included the same type of noninvasive therapy as the intervention group, the studies were excluded.

Two researchers (C.L. and J.X.) independently screened titles and abstracts for the initial assessment. Full-text articles were assessed independently by the two researchers to identify the final included articles. Disagreements were resolved by the third researcher (W.L.) in two stages. If the data were published in multiple studies, the most complete and effective analysis was included.

### Data extraction

To examine the effectiveness of noninvasive therapy interventions on pain intensity, pressure pain threshold, and pain-related disability of MTrPs, two researchers (C.L. and J.X.) independently performed the data extraction. Disagreements in data collection were solved by discussion with a third reviewer (W.L.). The extracted data of included studies consisted of (1) study characteristics (first author, year of publication, country, and sample size); (2) population characteristics (age, number of women, and location of muscle MTrPs); (3) intervention type; and (4) outcome measurements. Means and SDs of pain intensity, pressure pain threshold, and pain-related disability at the follow-up time point closest to the end of the treatment period were extracted. Due to differences in units, data were converted as required. Data were extracted from all study arms when the study included more than two types of noninvasive treatments independently and included in the NMA.

### Definition of terms

Myofascial pain refers to the common myofascial pain in the neck and lower back. In our study, due to no additional harm to patients, manual therapy, laser therapy, electronic therapy, extracorporeal shock wave therapy, ultrasound, exercise, medicine, Kinesio taping, heat, far-infrared ray, and combined therapy that included two or more than two types of therapy were listed as noninvasive therapies. Manual therapy was classified into the following categories: massage, ischemic compression, postisometric relaxation, biomechanical correction technique, self-myofascial release, and integrated neuromuscular inhibition technique. Laser therapy included high-level and low-level laser. The details of noninvasive therapeutic methods and comparisons are shown in Table 1.

**Table 1:** Interpretation of noninvasive therapeutic methods and comparisons.

Type of intervention	Interpretation
Manual therapy	
Massage	Gradually increasing the pressure through the finger for 5–10 s. The pressure volume did not exceed the pain pressure threshold for each patient
Ischemic compression	Putting fingers on the shock touch point could produce tolerable pain, and constant pressure. The pressure continued to increase and lasted for tens of seconds, and the patient felt tenderness and continued to repeat the process
Postisometric relaxation	The target muscle underwent a moderate isometric contraction
Biomechanical correction technique	Including postisometric, postreciprocal, antigravity relaxation of the muscles, myofascial release, and postisometric spinal auto-mobilization techniques
Self-myofascial release	Patients used a tool (e.g. a foam roller) to perform myofascial release exercises by themselves. They used own weight to pressure the soft tissues during exercise
Integrated neuromuscular inhibition technique	Including the combination of the ischemic compression technique, the strain-counter-strain technique, and the muscle energy technique
Laser therapy	Including both high-level and low-level lasers with a kind of laser instrument
Electronic therapy	It referred to the transcutaneous electrical nerve stimulation. Operation was used by a transcutaneous electrical stimulation instrument
Extracorporeal shock wave therapy	It referred to apply external shock wave instrument (e.g. Dornier AR2) to target muscle
Ultrasound	It referred to apply ultrasound therapy instrument (e.g. Sonoplus 992, Enraf-Nonius, and Delft)
Exercise	Including strengthening (isometric exercises, neck flexion, extension, right/left lateral flexion, pectoral muscles, the posterior part of the deltoids muscle, and posture exercises) and stretching (neck flexion, extension, right/left lateral flexion, right/left rotation, and pectoral muscle) exercises
Medicine	Including topical external medication (e.g. ketoprofen patches and topical capsaicin patches) and oral medication
Kinesio taping	Using Kinesio taping on targeted muscles
Heat	Using heating pad on targeted muscles
Far-infrared ray	Using a far-infrared device
Combined therapy	Including the above mentioned two or more therapies at the same time
Comparisons	One type of noninvasive therapy different from the intervention group, or no treatment, sham treatment, placebo treatment, control treatment

### Outcomes

The primary outcome measure was pain intensity [e.g. visual analog scale (VAS), numeric rating scale]. More specifically, pain intensity referred to the pain score using the relevant pain scale (i.e. VAS, numeric rating scale) after receiving a noninvasive therapy. After receiving a noninvasive treatment, the most recent pain intensity was measured as our outcome. The secondary outcomes were pressure pain threshold (i.e. algometer), as well as pain-related disability (e.g. neck disability index, neck pain and disability scale). The secondary outcomes were measured after the completion of the treatment.

### Risk of bias assessment

The risk of bias of included studies was independently assessed by two researchers (C.L. and J.X.) using the Cochrane Collaboration’s tool for assessing the risk of bias RoB 2^31^. A third reviewer (W.L.) was available to resolve disagreements as required. The tool consists of five domains, including (1) randomization process, (2) deviations from intended interventions, (3) missing outcome data, (4) measurement of the outcome, and (5) selection of the reported results. Low, moderate, or high bias risk were assigned to each domain and an overall risk bias score was determined on this basis.

### GRADE assessment

The Grading of Recommendations Assessment, Development and Evaluation (GRADE) approach was used to evaluate the quality of the evidence^32^. The following included study limitations, indirectness and transitivity, statistical heterogeneity and inconsistency, imprecision, and publication bias. Depending on the assessment of each of the factors mentioned above, the certainty of the evidence of the included studies was downgraded to moderate, low, or very low quality.

### NMA assumptions

Three assumptions were analyzed before conducting the NMA^33^. The first is similarity, which refers to the fact that baseline study characteristics should be similar and can be compared in studies included in the NMA. Similarity was assessed by examining whether the samples for each category of noninvasive intervention were similar in the baseline distribution of the variables affected (e.g. age, sex, basal pain, basal pressure pain threshold, and basal pain-related disability). The second is heterogeneity, which assumes that there should be no heterogeneity in the findings in studies with pairwise comparisons. Heterogeneity was tested using the *I*^2^ and *τ*^2^. The third is the inconsistency, which indicates that there are no relevant differences between direct and indirect evidence. Based on node-splitting, indirect and direct evidence were used to assess the existence of inconsistencies. With *P-*values, disagreement was statistically tested and reported^33^.

### Geometry of the network

The basic characteristics (age, sample size, interventions, outcome measures, etc.) of the studies included in the NMA are summarized in Table 2. For the whole NMA, we created a network graph for each result, in which nodes represented different interventions and the size of the nodes denoted the number of participants. In addition, the thickness of the edge line was considered the weight of the pairwise comparison sum. Visual forest plots were used to display the results of various noninvasive interventions compared to control.

**Table 2:** Characteristics of included studies.

References	Age, year (Mean± SD)	Sample size (female)	Intervention type	Intervention duration	MTrPs location	Outcome
Kiraly *et al*.^34^ (2018)	62.62±9.62	61 (54)	Laser	3 weeks	Trapezius	VAS and NDI
	57.26±14.31		Shock wave^a^			
Ahmed *et al*.^35^ (2020)	39.4±11.6	45 (0)	Manual	5 days	Upper trapezius muscle	VAS, PPT, and NDI
	38.7±13.3		Laser			
	38.4±13.3		Control^a^			
Rahbar *et al*.^36^ (2021)	38.09±9.67	72 (54)	Shock wave+neck stretching	4 weeks	Neck and upper back area	VAS, PPT, and NDI
	36.72±6.92		US+neck stretching			
	40.50±10.13		Neck stretching			
Yildirim *et al*.^37^ (2018)	29.8±5.2	54 (31)	US	NR	Trapezius muscle	VAS and PPT
	31.1±5.7		Placebo^a^			
Cabrera-Martos *et al*.^38^ (2022)	28.84±5.78	40 (30)	Manual	4 weeks	Neck	VAS
	32.50±4.68		Control^a^			
Lytras *et al*.^39^ (2020)	46.80±8.85	40 (30)	Therapeutic exercise+INIT	34 weeks	Neck	VAS, PPT, and NDI
	45.80±7.73		Therapeutic exercise^a^			
Taheri *et al*.^40^ (2021)	46.6±12.6	40 (28)	Shock wave	3 weeks	Neck	VAS and NDI
	48.5±12.1		Phonophoresis^a^			
Iaroshevskyi *et al*.^41^ (2019)	NR	87 (44)	Biomechanical correction of the musculoskeletal+therapeutic exercise	10 days	Neck	VAS
	NR		Therapeutic exercise^a^			
Ay *et al*.^42^ (2017)	44.80±17.19	73 (50)	KT	15 days	Upper neck and levator scapula muscle	VAS, PPT, and NPAD
	44.10±17.45		Sham^a^			
Taheri *et al*.^43^ (2016)	45.30±7.70	46 (43)	Laser+stretching exercises and medication	2 weeks	Upper trapezius	VAS and NDI
	42.30±10.40		Shock wave+stretching exercises and medication^a^			
Aktürk *et al*.^26^ (2018)	33.45±8.02	60 (40)	ESWT	2 weeks	MPS	VAS and PPT
	35.45±8.07		Sham ESWT^a^			
	35.65±11.03		US			
Azatcam *et al*.^44^ (2017)	41.56±9.50	89 (48)	TENS+stretching exercises	2 weeks	Trapezius	VAS, PPT, and NDI
	37.13±9.96		KT+stretching exercises			
	36.34±10.10		Stretching exercises^a^			
Kim *et al*.^45^ (2014)	44.76±12.71	99 (86)	NSAID patch^a^	2 weeks	Upper trapezius	NRS, PPT, and NDI
	49.17±13.52		NSAID patch+TENS			
	47.56±10.67		NSAID patch+HT			
	48.88±11.11		NSAID patch+CAP			
Dündar *et al*.^46^ (2015)	40.2±12.9	76 (76)	HILT + exercise	3 weeks	Trapezius	VAS and NDI
	38.4±12.1		Placebo HILT + exercise^a^			
Acar and Yilmaz^47^ (2012)	35.70±11.12	60 (51)	HT+ exercise	NR	Neck and upper back area	MPQ
	38.55±13.04		Exercise			
	37.50±10.45		Control^a^			
Lai *et al*.^48^ (2014)	53.9±11.2	48 (16)	FIR	1 week	MPS	VAS and PPT
	56.9±9.2		Control^a^			
Cho *et al*.^49^ (2012a)	47.67±10.49	36 (NR)	Stabilization exercises	NR	Upper Trapezius	VAS, PPT, and NDI
	47.06±13.53		ESWT			
	48.08±12.24		ESWT + stabilization exercises			
Cho *et al*.^50^ (2012b)	40.33±14.15	61 (52)	CAP patch	4 weeks	Trapezius	VAS and NDI
	42.22±11.91		Hydrogel patch^a^			
Kavadar *et al*.^51^ (2015)	37.43±9.07	59 (49)	US	NR	Trapezius	VAS and PPT
	35.83±5.68		Placebo^a^			
Sumen *et al*.^52^ (2015)	41.66±9.26	45 (32)	LLLT + stretching exercises	10 days	Upper trapezius	VAS, PPT, and NDI
	39.00±11.65		IMS + stretching exercises			
	35.26±11.70		Stretching exercises^a^			
Lai *et al*.^53^ (2017)	37.55±7.96	189 (129)	FIR	NR	Upper trapezius	VAS and PPT
	36.67±7.04		Placebo^a^			
Moraska *et al*.^54^ (2018)	28.4±6.7	25 (22)	Massage	NR	Upper trapezius	VAS and PPT
	29.7±6.6		Sham US^a^			
Rangon *et al*.^55^ (2018)	55.40±9.10	20 (20)	Ischemic compression + KT	5 weeks	Upper trapezius	NRS and PPT
	54.40±4.90		KT^a^			
Chao *et al*.^56^ (2016)	30.0±6.5	31 (28)	MPR^a^	7 days	Upper trapezius	VAS and PPT
	28.0±4.6		MPR + KT			
Kannan^57^ (2012)	32.00±9.33	45 (22)	Therapeutic US	5 days	Upper trapezius	VAS
	29.00±10.23		Laser			
	31.24±9.34		Ischemic compression			
Kim *et al*.^58^ (2016)	71.15±5.06	45 (40)	Self-exercise with a therapeutic inflatable ball	NR	Upper trapezius	VAS and PPT
	67.71±5.65		US			
Alayat *et al*.^59^ (2020)	28.47±5.07	50 (34)	Laser+PRT	4 weeks	Upper trapezius	VAS and PPT
	27.70±4.56		Sham laser + PRT^a^			
Mohammadi *et al*.^60^ (2016)	27.86±6.64	28 (28)	PRT	NR	Upper trapezius	VAS and PPT
	28.29±6.58		Control^a^			
Lin *et al*.^61^ (2012)	33.39±11.04	55 (51)	Lidocaine patch	1 week	Upper trapezius	VAS, PPT, and NDI
	36.19±12.34		Placebo patch^a^			
Öztürk *et al*.^62^ (2016)	29.95±4.90	37 (28)	KT	NR	Trapezius	VAS and PPT
	33.86±8.47		Sham^a^			
Bingölbali *et al*.^63^ (2023)	34.22±2.00	80 (55)	HT, TENS, and US^a^	4 weeks	Trapezius or levator scapulae	VAS and NPAD
	34.27±1.40		Massage +HT, TENS and US			
Rodriguez-Huguet *et al*.^64^ (2018)	38.24±9.06	41 (21)	MRT	2 weeks	Suboccipital and upper trapezius muscles	VAS and PPT
	37.80±8.75		US + TENS + massage			
Ibrahim *et al*.^65^ (2017)	23.61±4.11	30 (NR)	Shock wave	2 weeks	Trapezius	PPT and NDI
	25.64±5.39		Pressure release^a^			
Yildirim *et al*.^66^ (2016)	32.3±7.0	60 (45)	HT+TENS+US^a^	5 days	Trapezius or levator scapulae muscles	VAS, PPT, and NDI
	33.0±6.3		HT+TENS+US+manual therapy			
Kaur and Kapila^67^ (2017)	NR	20 (NR)	Massage	2 weeks	Upper trapezius muscles	NRS and NDI
	NR		US			
Gezgİnaslan *et al*.^68^ (2020)	45.0±12.0	94 (78)	ESWT	2 weeks	MPS	VAS and NDI
	43.3±11.9		Control^a^			
Kalichman *et al*.^69^ (2018)	25.44±1.63	30 (21)	KT	NR	Upper trapezius	PPT
	26.06±1.88		Control^a^			
Buttagat *et al*.^70^ (2016)	21.72±2.05	50 (43)	Massage	NR	Upper trapezius	VAS
	22.76±4.10		Control^a^			
Altan *et al*.^71^ (2005)	43.48±2.42	48 (32)	Laser therapy	2 weeks	NR	VAS and PPT
	43.32±2.10		Control^a^			
Mohamadi *et al*.^72^ (2017)	21.63±1.59	58 (NR)	Friction massage	3 days	Upper trapezius muscle	PPT
	22.04±1.76		Kinesio taping			

a Represents control group.

CAP, capsaicin; ESWT, extracorporeal shock wave therapy; FIR, far-infrared ray; HILT, high intensity laser therapy; HT, heat; IMS, intramuscular stimulation therapy; INIT, the integrated neuromuscular inhibition technique; KT, Kinesio taping; LLLT, low-level laser therapy; MPQ, McGill pain questionnaire; MPR, manual pressure release; MPS, myofascial pain syndrome; MRT, myofascial release therapy; MTrPs, myofascial trigger points; NDI, neck disability index; NPAD, neck pain and disability scale; NR, not reported; NRS, numerical rating scale; NSAID, nonsteroidal anti-inflammatory drugs; PPT, pressure pain threshold; PRT, pressure release technique; TENS, transcutaneous electrical nerve stimulation; US, ultrasound; VAS, visual analog scale.

### Statistical models

Three frequentist NMAs were performed to individually investigate pain intensity, pressure pain threshold, and pain-related disability. When interventions for pairwise comparisons exist, standard pairwise meta-analysis was used to comparing to intervention effects, and the estimation of effect sizes and 95% CIs was performed by the random effects DerSimonian-Laird method^73^.

As the results were continuous variables, mean difference (MD) was chosen as a standardized pooled effect size in pairwise comparisons. For interventions without direct comparison, a comparison of effect sizes was performed by indirect comparison^33^. The ranking of mixed effect sizes and 95% CIs for all combinations of therapies was presented using forest plots and league tables. Through a cumulative rank gram and the estimated surface under the cumulative ranking (SUCRA) for each intervention, the likelihood of each noninvasive treatment being the most effective method was presented. SUCRA implies that a numerical value between 0 (for the worst intervention) and 1 (for the best intervention) is assigned. Funnel plots were used to assess publication bias. Stata 16.0 (Stata Corporation, College Station), Revman 5.4 (Cochrane) were used for all statistical analyses.

## Results

### Study selection

**Figure 1:**
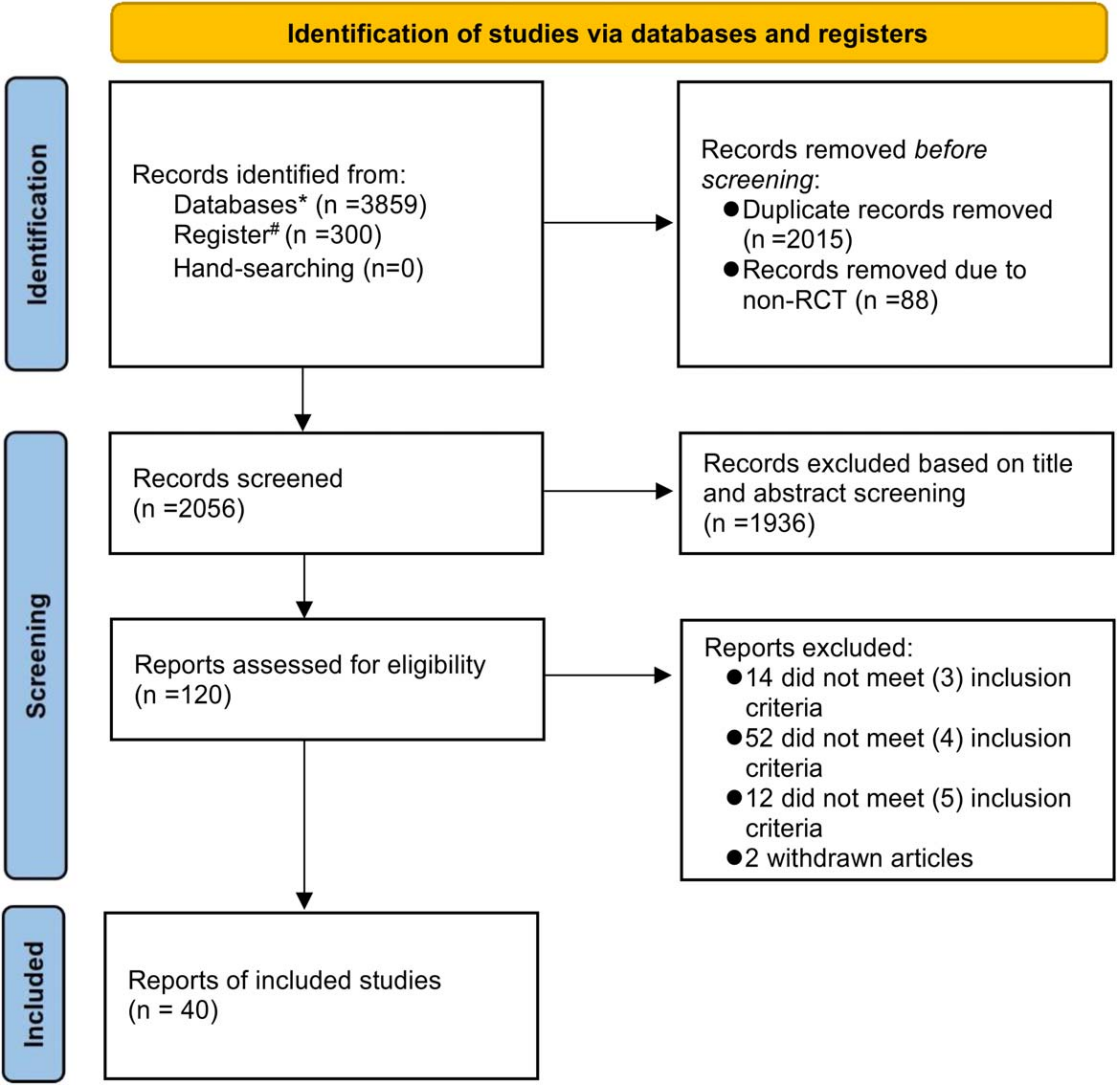
Flowchart of included studies. * Represents PubMed, Embase, CINAHL Complete, Web of Science, Scopus.^#^ Represents Cochrane.

Four thousand one hundred fifty nine records were initially obtained through the database search. The flowchart of study selection is presented in Figure 1. There were 40 RCTs included in the final NMA, involving a total sample of 2227 participants. In accordance with population (P), intervention (I), comparison (C), outcome (O), and study (S) principle, records that did not meet the requirements were excluded, including patient population (*n*=14), same types of intervention (*n*=52), missing or incorrect representation of the outcome (*n*=12), and withdrawn articles (*n*=2).

### Risk of bias assessment

In general, 29 (74.4%) records had a moderate risk of bias and only two had a low-risk of bias. Due to the implementation of different methods of intervention for patients, it was difficult to blind patients and operators. For this reason, many studies were rated as high risk of bias. In the selection of the reported result domain, 35 records were rated as moderate risk and five records were rated as low-risk. In the randomization process and measurement of the outcome domains, the majority of records was regarded as low-risk. The details are shown in Supplemental Materials 2 and 3 (Supplemental Digital Content 1, http://links.lww.com/JS9/B312).

### Study characteristics

Study characteristics of the included 40 records and 2227 participants in this NMA are shown in Table 2^26,34–72^. The majority of the patient population was female (67.85%), and the trapezius muscle was regarded as in position by the majority of records. The majority of records (*n*=31) were two-arm studies, and eight records were three-arm studies. Pain intensity was reported in 33 studies using the VAS tool, and only four articles used another pain scale tool to assess pain intensity. There were 26 and 19 records to report pressure pain threshold and pain-related disability, respectively. For pain-related disability, the majority of records (*n*=17) used the neck disability index and only two articles chose the neck pain and disability scale. Details of the allocation into treatment arms are shown in Supplemental Materials 4.1, 4.2, and 4.3 (Supplemental Digital Content 1, http://links.lww.com/JS9/B312). Baseline pain intensity, pressure pain threshold, pain-related disability, percentage of women, and patient age were similar across most treatment comparisons (Supplemental Material 5, Supplemental Digital Content 1, http://links.lww.com/JS9/B312).

### Primary outcome

**Figure 2:**
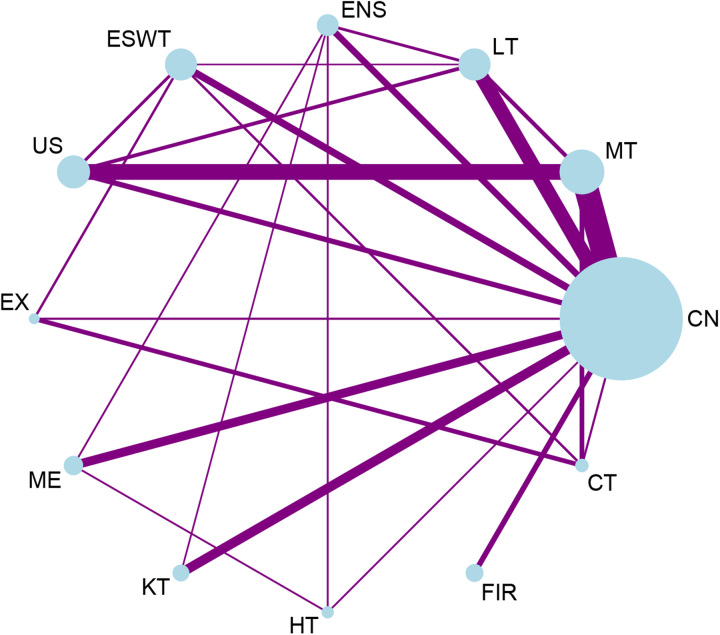
Network constructed for pain intensity. CN, control, sham, placebo; CT, combination therapy; ENS, electrical nerve stimulation; ESWT, extracorporeal shock wave therapy; EX, exercise; FIR, far-infrared ray; HT, heat; KT, Kinesio taping; LT, laser therapy; ME, medication; MT, manual therapy; US, ultrasound.

There were 37 studies with 2066 participants included in the NMA for pain intensity. There were 84 treatment arms in the network plot, including 11 therapeutic methods for pain intensity. As shown in Figure 2, most intervention methods made pairwise comparisons with the control group. Meanwhile, there were also contrasts between the two different methods of noninvasive intervention. The most studied interventions were manual therapy (*n*=297 participants), while placebo, sham, and control were used as the comparator arm in 28 studies (765 patients receiving placebo or control). The network plot further demonstrated that multi-arm studies with more than two types of intervention were included. However, there were intervention types with no direct contrast for some interventions, for example, ultrasound vs. exercise; exercise vs. medication; medication vs. Kinesio taping; Kinesio taping vs. heat. The most frequently performed comparison was manual therapy compared to the control group (nine groups).

### Secondary outcomes

**Figure 3:**
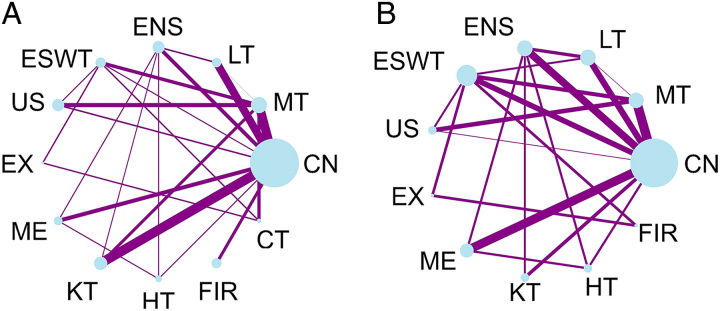
Network plots of treatment comparison for pressure pain threshold and pain-related disability. (A) Network of treatment comparison between interventions on pressure pain threshold. The size of the nodes represented the number of participants in different interventions. And the thickness of the edge line was considered the weight of pairwise comparison sum; (B) Network of treatment comparison between interventions on pain-related disability. The size of the nodes represented the number of participants in different interventions. And the thickness of the edge line was considered the weight of pairwise comparison sum. CN, control, sham, placebo; CT, combination therapy; ENS, electrical nerve stimulation; ESWT, extracorporeal shock wave therapy; EX, exercise; FIR, far-infrared ray; HT, heat; KT, Kinesio taping; LT, laser therapy; ME, medication; MT, manual therapy; US, ultrasound.

There were 26 studies included in the NMA for pressure pain threshold including 1393 participants and 58 study arms. As depicted in Figure 3A, pairwise comparisons of control, manual, and Kinesio taping were most frequently conducted (nine groups). As for intervention methods, manual therapy was performed most frequently, while a control group was present in 20 studies and had the largest number of participants (*n*=500).

For pain-related disability, there were 19 studies with 1079 participants and 45 study arms (see Fig. 3B). However, unlike the above results, this NMA included 10 types of intervention methods. For pain-related disability, the largest number of participants in noncontrol groups were extracorporeal shock wave therapy patients (*n*=172).

### Pairwise meta-analysis

Supplemental Material 6 (Supplemental Digital Content 1, http://links.lww.com/JS9/B312) shows the results of a pairwise comparison of two therapeutic methods. The results showed that manual therapy (MD of pain: −1.54, 95% CI: −2.44 to −0.64; MD of pressure pain threshold: 0.32, 95% CI: 0.08 to 0.57; MD of pain-related disability: −4.71, 95% CI: −7.20 to −2.22) was more effective than control in terms of pain intensity, pressure pain threshold, and pain-related disability. Laser (MD of pressure pain threshold: 0.96, 95% CI: 0.55 to 1.37) appeared to be associated with a more effective pressure pain threshold compared to control treatments. Moreover, extracorporeal shock wave therapy (MD of pain intensity: −2.10, 95% CI: −3.04 to −1.16; MD of pain-related disability: −8.61, 95% CI: −16.41 to −0.81) was more effective than control about pressure pain threshold and pain-related disability. Compared to control groups, electrical nerve stimulation (MD: −0.83, 95% CI: −1.33 to −0.34) and ultrasound (MD: −1.49, 95% CI: −2.26 to −0.73) were more efficacious for pain intensity. However, medication (MD of pain: −0.29, 95% CI: −0.64 to 0.05; MD of pressure pain threshold: 0.22, 95% CI: −0.16 to 0.59; MD of pain-related disability: −1.35, 95% CI: −2.89 to 0.19) did not differ from the control treatment in pain intensity, pressure pain threshold, and pain-related disability. As for pressure pain threshold and pain, there was no effect of the far-infrared ray (MD of pain: −0.11, 95% CI: −0.58 to 0.35; MD of pressure pain threshold: 0.04, 95% CI: −0.27 to 0.35) compared to controls. In terms of exercise, heat, combination therapy, only one study demonstrated their effects different than the control group. Except for controls, a noninvasive intervention was no different from any other method.

### Synthesis of results

**Figure 4:**
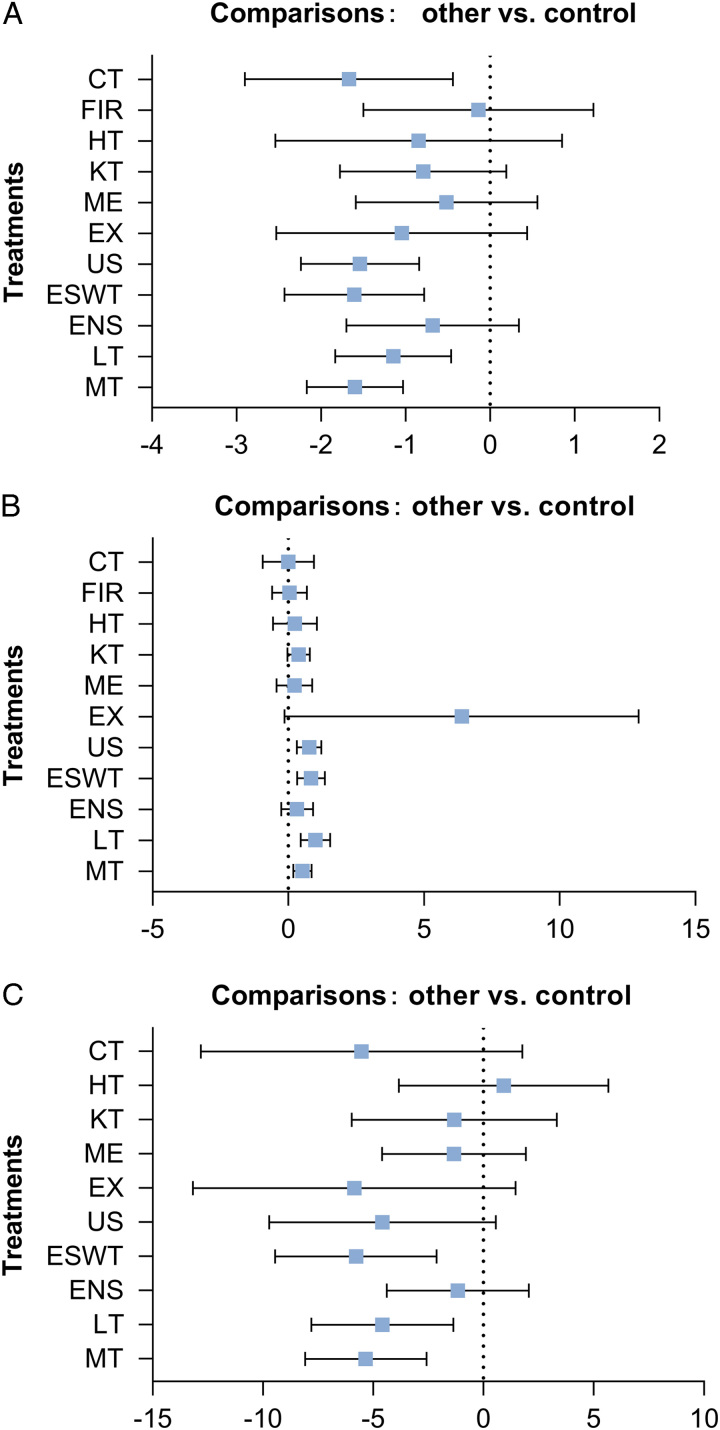
Forest plots for pain intensity, pressure pain threshold and pain-related disability. (A) Compared to control, the treatment effective of different methods for easing pain intensity; (B) Compared to control, the treatment effective of different methods for pressure pain threshold; (C) Compared to control, the treatment effective of different methods for pain-related disability. CAP, capsaicin; ESWT, extracorporeal shock wave therapy; FIR, far-infrared ray; HILT, high intensity laser therapy; HT, heat; IMS, intramuscular stimulation; INIT, the integrated neuromuscular inhibition technique; KT, Kinesio taping; LLLT, low-level laser therapy; MPR, manual pressure release; MRT, myofascial release therapy; NSAI, nonsteroidal anti-inflammatory; PRT, pressure release technique; TENS, transcutaneous electrical nerve stimulation; US, ultrasound.

As shown in Figure 4A, NMA showed that five methods, namely: manual therapy (MD: −1.60, 95% CI: −2.17 to −1.03, GRADE=low), laser therapy (MD: −1.15, 95% CI: −1.83 to −0.46, GRADE=low), extracorporeal shock wave therapy (MD: −1.61, 95% CI: −2.43 to −0.78, GRADE=low), ultrasound therapy (MD: −1.54, 95% CI: −2.24 to −0.84, GRADE=low), and combined therapy (MD: −1.67, 95% CI: −2.90 to −0.44, GRADE=low) were more effective in managing pain intensity compared to the control. For pressure pain threshold, the results in Figure 4B were similar to pain intensity, with only four interventions (manual therapy: 0.52, 95% CI: 0.19 to 0.86; laser therapy: 1.00, 95% CI: 0.46 to 1.54; extracorporeal shock wave therapy: 0.84, 95% CI: 0.33 to 1.35; ultrasound: 0.77, 95% CI: 0.31 to 1.22, GRADE=low) being more effective than the control group. However, manual therapy (MD: −5.34, 95% CI: −8.09 to −2.58, GRADE=low), laser therapy (MD: −4.58, 95% CI: −7.80 to −1.36, GRADE=very low), and extracorporeal shock wave therapy (MD: −5.78, 95% CI: −9.45 to −2.12, GRADE=very low) were more effective than the controls in managing pain-related disability (Fig. 4C).

As shown in Table 3, it was concluded that no differences were found between the two types of intervention for pain intensity from the league table, which presented the comparative effects for all interventions. However, laser therapy (MD: 0.96, 95% CI: 0.12 to 1.80, GRADE=low) had a higher score than far-infrared ray for the pressure pain threshold as well as a variety of noninvasive intervention methods. For pain-related disability, the comparison of noninvasive methods showed that manual therapy (MD: −6.26, 95% CI: −11.74 to −0.79, GRADE=moderate), and extracorporeal shock wave therapy (MD: −6.70, 95% CI: −12.70 to −0.71, GRADE=low) had a lower disability index compared to heat, as shown in Supplemental Material 7 (Supplemental Digital Content 1, http://links.lww.com/JS9/B312).

**Table 3:** League table reporting the comparative effects for all interventions for the pain intensity network and pressure pain threshold.

Pressure pain threshold											
Pain intensity											
MT	−0.48 (−1.06, 0.11)	0.20 (−0.46, 0.86)	−0.31 (−0.84, 0.21)	−0.24 (−0.73, 0.25)	−5.86 (−12.39, 0.66)	0.30 (−0.44, 1.03)	0.14 (−0.33, 0.61)	0.28 (−0.59, 1.16)	0.48 (−0.24, 1.21)	0.52 (−0.37, 1.41)	**0.52** (**0.19, 0.86)**
−0.46 (−1.26, 0.35)	LT	0.68 (−0.11, 1.46)	0.16 (−0.56, 0.88)	0.23 (−0.45, 0.92)	−5.39 (−11.93, 1.16)	0.77 (−0.07, 1.62)	0.62 (−0.03, 1.27)	0.76 (−0.21, 1.73)	**0.96** (**0.12, 1.80)**	1.00 (−0.07, 2.06)	**1.00** (**0.46, 1.54)**
−0.92 (−2.07, 0.23)	−0.46 (−1.63, 0.70)	ENS	−0.52 (−1.29, 0.26)	−0.44 (−1.18, 0.29)	−6.06 (−12.61, 0.49)	0.10 (−0.68, 0.88)	−0.06 (−0.69, 0.57)	0.08 (−0.79, 0.96)	0.28 (−0.59, 1.15)	0.32 (−0.79, 1.43)	0.32 (−0.26, 0.91)
0.00 (−0.92, 0.92)	0.46 (−0.47, 1.39)	0.92 (−0.37, 2.21)	ESWT	0.07 (−0.47, 0.62)	−5.55 (−12.07, 0.97)	0.61 (−0.22, 1.44)	0.46 (−0.17, 1.09)	0.60 (−0.36, 1.56)	0.80 (−0.02, 1.62)	0.84 (−0.20, 1.87)	**0.84** (**0.33, 1.35)**
−0.06 (−0.79, 0.67)	0.40 (−0.48, 1.28)	0.86 (−0.36, 2.08)	−0.06 (−0.97, 0.84)	US	−5.62 (−12.15, 0.91)	0.54 (−0.26, 1.33)	0.38 (−0.21, 0.97)	0.53 (−0.40, 1.45)	0.72 (−0.06, 1.51)	0.76 (−0.25, 1.78)	**0.77** (**0.31, 1.22)**
−0.55 (−2.08, 0.97)	−0.10 (−1.68, 1.49)	0.37 (−1.42, 2.16)	−0.56 (−2.05, 0.93)	−0.49 (−2.07, 1.08)	EX	6.16 (−0.40, 12.72)	6.01 (−0.53, 12.54)	6.15 (−0.43, 12.72)	6.35 (−0.21, 12.90)	6.39 (−0.14, 12.91)	6.39 (−0.14, 12.91)
−1.09 (−2.30, 0.13)	−0.63 (−1.89, 0.63)	−0.17 (−1.50, 1.17)	−1.09 (−2.44, 0.26)	−1.03 (−2.31, 0.25)	−0.53 (−2.36, 1.30)	ME	−0.16 (−0.91, 0.60)	−0.01 (−0.87, 0.84)	0.19 (−0.73, 1.10)	0.22 (−0.93, 1.38)	0.23 (−0.43, 0.88)
−0.81 (−1.95, 0.33)	−0.35 (−1.54, 0.84)	0.11 (−1.16, 1.38)	−0.81 (−2.09, 0.47)	−0.75 (−1.96, 0.46)	−0.26 (−2.03, 1.52)	0.28 (−1.16, 1.71)	KT	0.14 (−0.74, 1.03)	0.34 (−0.42, 1.10)	0.38 (−0.63, 1.39)	0.38 (−0.03, 0.79)
−0.75 (−2.54, 1.03)	−0.30 (−2.11, 1.52)	0.17 (−1.58, 1.92)	−0.76 (−2.64, 1.12)	−0.69 (−2.52, 1.14)	−0.20 (−2.45, 2.05)	0.33 (−1.43, 2.10)	0.06 (−1.86, 1.98)	HT	0.20 (−0.83, 1.23)	0.24 (−1.01, 1.49)	0.24 (−0.57, 1.05)
−1.46 (−2.93, 0.02)	−1.00 (−2.52, 0.52)	−0.54 (−2.24, 1.16)	−1.46 (−3.05, 0.13)	−1.40 (−2.93, 0.13)	−0.90 (−2.92, 1.11)	−0.37 (−2.11, 1.36)	−0.65 (−2.33, 1.03)	−0.71 (−2.88, 1.47)	FIR	0.04 (−1.11, 1.19)	0.04 (−0.60, 0.68)
0.07 (−1.18, 1.31)	0.52 (−0.83, 1.88)	0.99 (−0.60, 2.58)	0.06 (−1.24, 1.37)	0.13 (−1.21, 1.46)	0.62 (−0.81, 2.05)	1.15 (−0.48, 2.79)	0.88 (−0.70, 2.45)	0.82 (−1.27, 2.91)	1.53 (−0.31, 3.36)	CT	0.00 (−0.95, 0.95)
−**1.60** (−**2.17**, −**1.03)**	−**1.15** (−**1.83**, −**0.46)**	−0.68 (−1.70, 0.34)	−**1.61** (−**2.43**, −**0.78)**	−**1.54** (−**2.24**, −**0.84)**	−1.05 (−2.53, 0.44)	−0.52 (−1.59, 0.56)	−0.79 (−1.78, 0.19)	−0.85 (−2.54, 0.85)	−0.14 (−1.50, 1.22)	−**1.67** (−**2.90**, −**0.44)**	**CN**

Date is presented by mean difference with 95% CI. Bold denotes statistical significance at *P*<0.05.

CN, control; CT, combination therapy; ENS, electrical nerve stimulation; ESWT, extracorporeal shock wave therapy; EX, exercise; FIR, far-infrared ray; HT, heat pack; KT, Kinesio taping; LT, laser therapy; ME, medication; MT, manual therapy; US, ultrasound.

**Figure 5:**
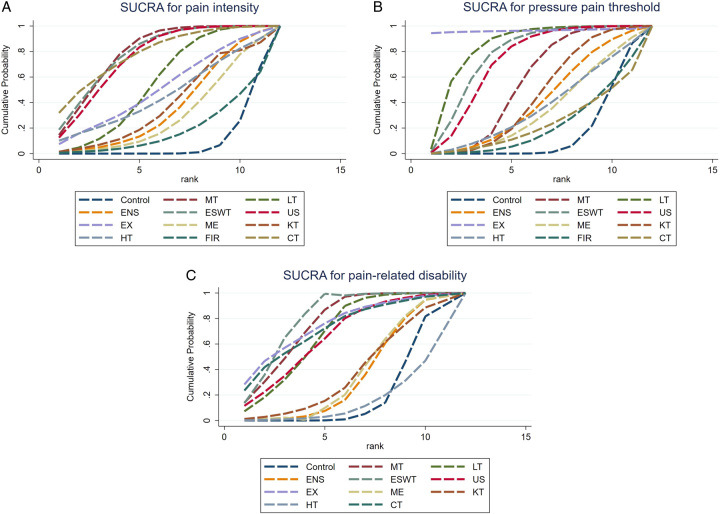
Surface under the cumulative ranking (SUCRA) for pain intensity, pressure pain threshold, and pain-related disability. (A) The cumulative probability of noninvasive methods of being the most effective in easing pain intensity; (B) The cumulative probability of noninvasive methods of being the most effective in pressure pain threshold; (C) The cumulative probability of noninvasive methods of being the most effective in relieving pain-related disability. CN, control, sham, placebo; CT, combination therapy; ENS, electrical nerve stimulation; ESWT, extracorporeal shock wave therapy; EX, exercise; FIR, far-infrared ray; HT, heat; KT, Kinesio taping; LT, laser therapy; ME, medication; MT, manual therapy; US, ultrasound.

As shown in Figure 5A, the highest probabilities of the most effective treatment for pain intensity were combined therapy (32%) and its area under the curve was 78.3%. However, the method with the biggest area under the curve was manual therapy (79.4%), followed by extracorporeal shock wave therapy and combined therapy, while the control group was the worst in this regard. Manual therapy had the highest mean rank (3.3), followed by the mean rank of extracorporeal shock wave therapy, which was 3.4, the same as combined therapy. And the control group had the lowest mean rank^11^. For the pressure pain threshold, the effective method of highest probabilities was exercise, followed by extracorporeal shock wave therapy and laser therapy (Fig. 5B). The mean rank of exercise was first (1.4), and the control group placed last (10.3). For pain-related disability, the area under the curve of extracorporeal shock wave therapy was the biggest (78.9%) and the mean rank was the first (3.1, Fig. 5C).

### Exploration for inconsistency

Supplemental Materials 8.1, 8.2, and 8.3 (Supplemental Digital Content 1, http://links.lww.com/JS9/B312) indicated that the direct estimate of the overwhelming majority of comparisons was consistent with the indirect estimate in the node-splitting analysis of pain intensity, pressure pain threshold and pain-related disability. As for the global inconsistency analysis, the *P*-value indicated that there was no inconsistency in pain intensity, pressure pain threshold, and pain-related disability (Supplemental Material 8.4, Supplemental Digital Content 1, http://links.lww.com/JS9/B312).

### Risk of bias across studies

Supplemental Materials 9.1, 9.2, and 9.3 (Supplemental Digital Content 1, http://links.lww.com/JS9/B312) display the certainty of the three-network evidence, including study limitations, indirection, inconsistency, imprecision, and publication bias. The mainly downgrade reasons were study limitation and imprecision. To assess whether the network plots were symmetrical, funnel plots were visually inspected. As illustrated in the funnel plots (Supplemental Materials 10.1, 10.2, and 10.3, Supplemental Digital Content 1, http://links.lww.com/JS9/B312), combined with the *P*-value of the Egger tests, the plots of pain intensity (*P*=0.088) and pressure pain threshold (*P*=0.135) were symmetrical, indicating no publication bias for pain intensity, and pressure pain threshold.

## Discussion

This is the first meta-analysis that compare the effectiveness of a variety of noninvasive interventions for myofascial pain. Although the evidences indicated that no differences were found in the comparisons of noninvasive methods for relieving pain^65,74^, our NMA suggested that manual, laser, extracorporeal shock wave therapy, ultrasound, and combination therapies were effective in relieving pain intensity when compared to the control group. In addition, the result of the pressure pain threshold was similar to the pain intensity, while manual, laser, extracorporeal shock wave therapy, and ultrasound therapies were more effective than placebo treatments. Meanwhile, our results showed that laser therapy was more effective than far-infrared ray in the comparison of noninvasive methods. For pain-related disability, we found that extracorporeal shock wave therapy and manual therapy were more effective compared to control and heat therapy. These results may help clinicians to choose appropriate noninvasive treatment modalities for patients with myofascial pain. For example, manual, laser, and extracorporeal shock wave therapy therapies exhibited effectiveness in pain relief, raising the pressure pain threshold, and reducing the pain disability simultaneously, which suggested that any of three treatments could be prioritized to relieve three symptoms at the same time. However, according to the GRADE assessment, the evidences were moderate to low so that interpretation of the outcomes should be taken with caution.

This study pooled the efficacy of various noninvasive intervention methods. Several noninvasive methods presented similar effects on relieving pain intensity. However, the minimal clinically important difference (MCID) of relieving pain intensity varied widely in different chronic pain models. A meta-analysis by conducted Olsen *et al*. indicated that MCID was a median of 20 mm in VAS among various chronic pain diseases. For example, the mean changes for MCID reached 19 mm for neck pain and 22 mm for inflammatory rheumatic pain^75^. However, a study assessing musculoskeletal pain intensity, reported the mean change of MCID of 14 mm^76^. Therefore, compared to control, pain score of manual therapy, laser therapy, extracorporeal shock wave therapy, ultrasound, and combination therapy decreased by 1.60, 1.15, 1.61, 1.54, and 1.67, respectively, which had some clinical significance.

Our study indicated that extracorporeal shock wave therapy was beneficial to easing pain. Extracorporeal shock wave therapy has been used for musculoskeletal disorders since 1990s^77^. The waves in extracorporeal shock wave therapy, formed with electromagnetic, piezoelectric, and electrohydraulic methods increase the production of prostaglandins and further improve tissue regeneration, and play a positive role in soft tissue inflammatory diseases such as fasciitis and tendinitis^34^. Extracorporeal shock wave therapy could relief pain by regulating the biological mechanisms of pain, inflammation, and angiogenesis in MPS and produce desensitization to the therapeutic area^7,78^. Our results are similar with other findings^28,79^, as extracorporeal shock wave showed no advantages over other noninvasive methods. However, compared to sham extracorporeal shock wave therapy and other treatments, standardized mean difference of extracorporeal shock wave therapy were 1.29 and 1.81, respectively^80^. It indicated that extracorporeal shock wave therapy was not only more effective than sham extracorporeal shock wave therapy, but also more effective than other noninvasive intervention methods such as ultrasound^80^. According to Rahbar *et al*., mean change of pain intensity was 1.77 for the extracorporeal shock wave therapy group and 1.20 for the ultrasound group, which suggested that extracorporeal shock wave therapy was more effective than the ultrasound^36^. Another study conducted by Taheri *et al*. also demonstrated that the effectiveness of the extracorporeal shock wave therapy on pain intensity was more beneficial than ultrasound (mean pain score of extracorporeal shock wave therapy group was 5.5, while the mean pain score of ultrasounds was 5.9)^40^. However, extracorporeal shock wave therapy did not differ in efficacy from other noninvasive treatments in this study. The reason could be that the results of this study were derived from the combination of multiple previous comparisons of ultrasound with extracorporeal shock wave therapy.

Our results also support the conclusion that manual therapy is effective when compared to the control group. This result was identical to a meta-analysis result that manual therapy showed a reduction of approximately1.75 points in pain intensity, where manual therapy was recommended as an effective strategy for pain relief^24^. One of the possible mechanisms of relieving pain intensity with manual therapy can be the inactivation of MTrPs, and relaxation of the constantly contracting muscles^13^. Another mechanism is related to improvements in blood circulation in the treated area, elimination of pain metabolizing substances, leading to further pain relief^24^.

This NMA suggested that ultrasound could relieve myofascial pain. Ultrasound could provide a thermal effect, which could promote vascular dilation, improve blood flow in the therapeutic area, and reduce the formation of pain-causing substances (e.g. bradykinin). It also could reduce muscle spasms and increase the growth capacity of collagen fibers^37^. For example, one meta-analysis demonstrated that compared to sham ultrasound, the percentage of improvement for pain was 9.6%^81^. Therefore, ultrasound could relieve pain caused by arthritis, which is similar to our findings^81^.

Moreover, laser therapy could also relieve musculoskeletal pain as a noninvasive treatment. Studies have reported that low-level laser therapy could cause the changes in cells and tissues, such as regulating cell metabolism, reducing inflammation, and improving blood circulation^46,82,83^. This study showed that there was no difference in effectiveness between laser therapy and other noninvasive treatments; however, laser therapy was more effective than the control group, which was consistent with the results of Momenzadeh *et al*.^84^. Meanwhile, in another meta-analysis, low-level laser therapy was shown to relieve the intensity of muscle pain (MD: −1.29, 95% CI: −2.36 to −0.23)^22^; however, it also showed that the therapeutic effectiveness laser therapy on MPS was far lower in other musculoskeletal disorders^85^.

From the point of view of certainty of evidence, for comparison with the control group, combined therapy had the widest CI, indicating a lack of precision in comparison to the control. However, manual therapy had a relatively narrow CI proving some certainty in the result for this treatment method. And the difference of CIs between combined therapy and other significant methods could be the result of a small sample in the included studies of combined therapy.

The findings of the pressure pain threshold were similar to pain intensity. But manual therapy, laser therapy, extracorporeal shock wave therapy, and ultrasound had narrow CIs providing some certainty in the results for these treatments methods. However, physical exercise had a wide CI, which could be related to the small sample size and small number of studies. And the physical exercise method had the largest effect value, which might result in the highest probability of physical exercise being the best treatment, whereas in fact exercise was not statistically significant compared to other noninvasive methods. It was noteworthy that evidence of quality was low or very low. Therefore, the result might not have proven force. A NMA conducted by Guzman *et al*.^24^ showed that afferent reduction techniques had the highest effect size for pressure pain threshold (0.93, 95% CI: 0.4–1.39), which indicated that manual therapy was regarded as an effective method to treat pressure pain threshold of MTrPs. However, a meta-analysis indicated that although laser therapy might decrease the sensitiveness of MTrPs on the pressure, it was necessary that considered stability of the pain threshold on muscles of different sites^25^. According to Jørgensen *et al*.^85^ study, the MCID of the pressure pain threshold was 0.48 on the fifth cervical vertebra for neck pain. Compared to control, manual therapy, laser therapy, extracorporeal shock wave therapy and ultrasound were improved by 0.52, 1.00, 0.84, 0.77, respectively. However, the measurement of the position might be different. Therefore, the clinical significance of this NMA result needs to be further explored.

This NMA showed that significant differences compared to manual therapy, laser therapy, extracorporeal shock wave therapy with the control group was existed, and the effects of these methods had relatively narrow CIs. However, heat therapy had wide CIs compared with other noninvasive methods, which might be related to these evidences that were from a small number of pairwise comparisons and small samples. A meta-analysis indicated that manual therapy improved function in the short term, which was similar to this NMA^86^. And according to a study conducted by Young *et al.*^87^, the MCID of the NDI score was regarded as 5.5 points. Therefore, this NMA indicated that these therapies were clinically significant in improving function compared to control. But it was not negligible that the quality of the evidence for the result of comparisons in this NMA was low.

This study synthesized several noninvasive therapies currently applied in patients with myofascial pain. More than two different methods could be compared in a quantitative manner through the NMA to provide information for clinical staff to make decisions. And the effectiveness of different methods is considered by the direct and indirect effects through conducting the NMA. However, this NMA have several limitations. Firstly, the quality of some evidences is low. It may be caused by study themselves. Because different intervention methods need to be implemented for the patients in the included studies, and it is difficult to blind the patients. Secondly, some results are based on several small-size studies, such as Kinesio taping versus electrical nerve stimulation. Thirdly, the clinical significance of some methods was small, so we should be caution when choosing appropriate therapeutic methods. Therefore, it is indispensable that RCTs of high quality are conducted to explore the efficacy of difference noninvasive methods on pain intensity, pressure pain threshold, and pain-related disability.

## Conclusion

In this NMA, manual therapy, laser therapy, and extracorporeal shock wave therapy could effectively reduce pain intensity, pressure pain threshold, and pain-related disability with statistical significance when compared with placebo. Additionally, laser therapy is more effective on increasing pressure pain threshold compared to far-infrared ray, and manual therapy and extracorporeal shock wave therapy are more effective than heat treatment in enhancing function. Moreover, the combination of manual therapy, extracorporeal shock wave therapy, laser therapy, and other methods can contribute to functional recovery. Our findings may provide clinicians with appropriate therapeutic modalities for patients with MPS among different scenarios.

## Ethical approval

Ethical declaration not applicable. This study is based on published literature.

## Consent

This study is based on published research and does not involve the patient’s personal privacy.

## Sources of funding

This study was supported by Study Project for Reformation of Undergraduate Teaching in Shandong Province (Grant Z2022107), Study Abroad Program for Shandong Provincial Government Education System (Grant 2022-44), and Overseas Study Fund Program for Shandong First Medical University (Shandong Academy of Medical Sciences).

## Conflicts of interest disclosure

The authors declare that they have no conflicts of interest.

## Author contribution

C.L. and Y.W.: wrote paper; G.D.: acquired of the financial support for the project leading to this publication, contributed and approved the final version of the manuscript; W.L. and W.Y.: conceived the content and design of the study; C.L. and J.X.: conducted the analyses.

## Research registration unique identifying number (UIN)

1. Name of the registry: PROSPERO.
2. Unique identifying number or registration ID: CRD42023400783.
3. Hyperlink to our specific registration: https://www.crd.york.ac.uk/prospero/display_record.php?ID=CRDXXX.

## Guarantor

The guarantor is Prof. Weihua Liu.

## Data availability statement

All data generated or analyzed during this study are included in this published article and its supplementary information files. All data in this manuscript is available and transparent for readers. Further inquiries (e.g. analytic code or any other materials) can be directed to the corresponding authors.

## Provenance and peer review

Not commissioned, externally peer-reviewed.

## Supplementary Material
