# Supplementary material for: Comparative effectiveness of noninvasive therapeutic interventions for myofascial pain syndrome: a network meta-analysis of randomized controlled trials
Source: Int J Surg. 2023 Nov 7;110(2):1099–112. doi: 10.1097/JS9.0000000000000860 (PMC10871620; doi:10.1097/JS9.0000000000000860)
Supplement: Supplementary file 1 [file js9-110-1099-s001.docx]

**Supplemental Digital Content 1**

**Comparative effectiveness of non-invasive therapeutic interventions for myofascial pain syndrome：a network meta-analysis of randomized controlled trials.**

Chang Liu, Yang Wang, Wenli Yu, Junai Xiang, Guoyong Ding, Weihua Liu

**Supplemental Tables and Figures**

| **Contents** | **Page** |
| --- | --- |
| **Supplementary material 1** Electronic database searching strategy | 4-7 |
| **Supplementary material 2** Risk of bias for the included studies | 8 |
| **Supplementary material 3** Risk of bias assessed with risk of bias tool for included studies | 9 |
| **Supplementary material 4.1** Summary table of all data included in the network for pain intensity | 10-14 |
| **Supplementary material 4.2** Summary table of all data included in the network for pressure pain threshold | 15-18 |
| **Supplementary material 4.3** Summary table of all data included in the network for pain-related disability | 19-21 |
| **Supplementary material 5** Similarity comparison of treatment. | 22-23 |
| **Supplementary material 6** Pairwise comparisons of meta-analysis of pain intensity, pressure pain threshold and pain-related disability | 24-31 |
| **Supplementary material 7** League table reporting the comparative effects for all interventions for pain-related disability | 32-33 |
| **Supplementary material 8.1** Node-splitting analysis of pain intensity | 34-35 |
| **Supplementary material 8.2** Node-splitting analysis of pressure pain threshold | 36 |
| **Supplementary material 8.3** Node-splitting analysis of pain-related disability | 37 |
| **Supplementary material 8.4** Global Wald test for inconsistency models | 38 |
| **Supplementary material 9.1** GRADE assessment for all pairwise comparisons within the pain intensity network | 39-40 |
| **Supplementary material 9.2** GRADE assessment for pairwise comparisons within the pressure pain threshold network | 41 |
| **Supplementary material 9.3** GRADE assessment for pairwise comparisons within the pain-related disability network | 42-43 |
| **Supplementary material 10.1** Funnel plot for pain intensity | 44 |
| **Supplementary material 10.2** Funnel plot for pressure pain threshold | 45 |
| **Supplementary material 10.3** Funnel plot for pain-related disability | 46 |
| **Supplementary material 11** Summary of changes between original protocol and final manuscript | 47-48 |

**Supplementary material 1.** Electronic databases searching strategy

| **Database** | **Step** | **Searching strategy** | **Number of articles** |
| --- | --- | --- | --- |
| PubMed | #1 | "Myofascial Pain Syndromes"[Mesh] | 6821 |
|  | #2 | "Trigger Points"[Mesh] | 786 |
|  | #3 | ((((myofascial pain [Title/Abstract])) OR (myofascial trigger points [Title/Abstract])) OR (myofascial*[Title/Abstract])) OR (trigger point*[Title/Abstract]) | 6580 |
|  | #4 | (((((((((((((((treat*[Title/Abstract])) OR (treatment[Title/Abstract])) OR (manual therapy[Title/Abstract])) OR (manual therap* [Title/Abstract])) OR (laser therapy[Title/Abstract])) OR (electronic therapy[Title/Abstract])) OR (extracorporeal shock wave therapy[Title/Abstract])) OR (ultrasound[Title/Abstract])) OR (exercise[Title/Abstract])) OR (sport*[Title/Abstract])) OR (medicine[Title/Abstract])) OR (medication[Title/Abstract])) OR (kinesio taping[Title/Abstract])) OR (hot pack[Title/Abstract])) OR (far-infrared irradiation[Title/Abstract])) | 7644772 |
|  | #5 | ((((random*[Publication Type])) OR (random* controlled trail [Publication Type])) OR (RCT [Publication Type])) OR ((((random*[Title/Abstract])) OR (random* controlled trail [Title/Abstract])) OR (RCT[Title/Abstract])) | 1545490 |
|  | #6 | (#1 OR #2 OR #3) AND #4 AND #5 | 965 |
| Embase | #1 | 'Myofascial pain'/exp OR 'trigger point'/exp | 10715 |
|  | #2 | 'myofascial pain':ab,ti OR 'myofascial trigger points':ab,ti OR myofascial*:ab,ti OR 'trigger point*':ab,ti | 8756 |
|  | #3 | 'randomized controlled trial':ab,ti OR 'randomized control trial':ab,ti | 141422 |
|  | #4 | 'manipulative medicine’: ab,ti OR 'laser therapy':ab,ti OR 'electrical nerve stimulation':ab,ti OR 'shock wave therapy':ab,ti OR ultrasound:ab,ti OR exercise:ab,ti OR sport:ab,ti OR medicine:ab,ti OR 'drug therapy':ab,ti OR 'kinesio taping':ab,ti OR heat:ab,ti OR 'far infrared radiation':ab,ti | 2083686 |
|  | #5 | #1 AND #2 AND #3 AND #4 | 62 |
| CINAHL Complete | S1 | SU Myofascial pain syndrome OR SU trigger points | 1066 |
|  | S2 | TI myofascial pain OR AB myofascial pain OR TI myofascial trigger points OR AB myofascial trigger points | 881 |
|  | S3 | TI intervention OR AB intervention OR TI treat * OR AB treat * OR TI manual therapy OR AB manual therapy OR TI laser therapy OR AB laser therapy OR TI electronic therapy OR AB electronic therapy OR TI extracorporeal shock wave therapy OR AB extracorporeal shock wave therapy | 895758 |
|  | S4 | TI ultrasound OR AB ultrasound OR TI exercise OR AB exercise OR TI sport* OR AB sport* OR TI medicine OR AB medicine OR TI kinesio taping OR AB kinesio taping OR TI hot pack OR AB hot pack | 1670825 |
|  | S5 | TI rct or randomized control trial or randomized controlled trial | 33215 |
|  | S6 | S1 OR S2 | 1371 |
|  | S7 | S3 OR S4 | 2502643 |
|  | S8 | S6 AND S7 AND S5 | 49 |
| Web of Science | #1 | (((((TS= (Myofascial pain syndrome)) OR TS= (trigger points)) OR TI= (myofascial pain)) OR AB=(myofascial pain)) OR TI=(myofascial trigger points)) OR AB=(myofascial trigger points) | 27354 |
|  | #2 | (((((((((((((((((((((TI=(treatment)) OR AB=(treatment)) OR TI=(treat*)) OR AB=(treat*)) OR TI=(manual therapy)) OR AB=(manual therapy)) OR TI=(laser therapy)) OR AB=(laser therapy)) OR TI=(electronic therapy)) OR AB=(electronic therapy )) OR TI=(extracorporeal shock therapy )) OR AB=(extracorporeal shock therapy)) OR TI=(ultrasound)) OR AB=(ultrasound)) OR TI=(sport*)) OR AB=(sport*)) OR TI=(medicine)) OR AB=(medicine)) OR TI=(kinesio taping )) OR AB=(kinesio taping OR hot pack)) OR TI=(hot pack)) OR AB=(hot pack) | 6354306 |
|  | #3 | (((((TI=(random*)) OR AB= (random*)) OR TI=(RCT)) OR AB=(RCT)) OR TI= (random controlled trail)) OR AB= (random controlled trail) | 1640998 |
|  | #4 | #1 AND #2 AND #3 | 1292 |
| Cochrane | #1 | MeSH descriptor: [Myofascial Pain Syndromes] this term only | 1111 |
|  | #2 | MeSH descriptor: [Trigger Points] this term only | 227 |
|  | #3 | (Myofascial pain): ti,ab,kw OR (Myofascial trigger points):ti,ab,kw | 2993 |
|  | #4 | (treatment): ti,ab,kw OR (treat*):ti,ab,kw OR ("extracorporeal shock wave therapy"):ti,ab,kw OR ("ultrasound"):ti,ab,kw OR ("exercise"):ti,ab,kw | 1091167 |
|  | #5 | (sports): ti,ab,kw OR (Medicine):ti,ab,kw OR (hot pack):ti,ab,kw OR (manual therapy):ti,ab,kw OR (laser therapy):ti,ab,kw | 107981 |
|  | #6 | (electronic therapy): ti,ab,kw | 0 |
|  | #7 | ("randomized control trial"): ti,ab,kw OR ("randomized clinical trials"):ti,ab,kw OR ("randomized control trials"):ti,ab,kw OR ("randomized clinical trial"):ti,ab,kw OR ("randomized controlled clinical trial"):ti,ab,kw | 94178 |
|  | #8 | (#1 OR #2 OR #3) AND (#4 OR #5 OR #6) AND #7 | 300 |
| Scopus | #1 | TITLE-ABS (myofascial AND pain AND syndrome) OR TITLE-ABS (trigger AND points) OR TITLE-ABS (myofascial AND pain) OR TITLE-ABS (myofascial AND trigger AND points) | 22900 |
|  | #2 | TITLE-ABS (treatment) OR TITLE-ABS (treat*) OR TITLE-ABS (manual AND therapy) OR TITLE-ABS (laser AND therapy) OR TITLE-ABS (electronic AND therapy) OR TITLE-ABS (extracorporeal AND shock AND wave AND therapy) OR TITLE-ABS (ultrasound) OR TITLE-ABS (exercise) OR TITLE-ABS (sport*) OR TITLE-ABS (medicine) OR TITLE-ABS (kinesio AND taping) OR TITLE-ABS (hot AND pack) OR TITLE-ABS (far-infrared AND irradiation) | 11507995 |
|  | #3 | TITLE-ABS (random*) OR TITLE-ABS (rct) OR TITLE-ABS (randomized AND controlled AND trail) OR TITLE-ABS (randomized AND control AND trail) | 2636179 |
|  | #4 | #1 AND #2 AND #3 | 1491 |


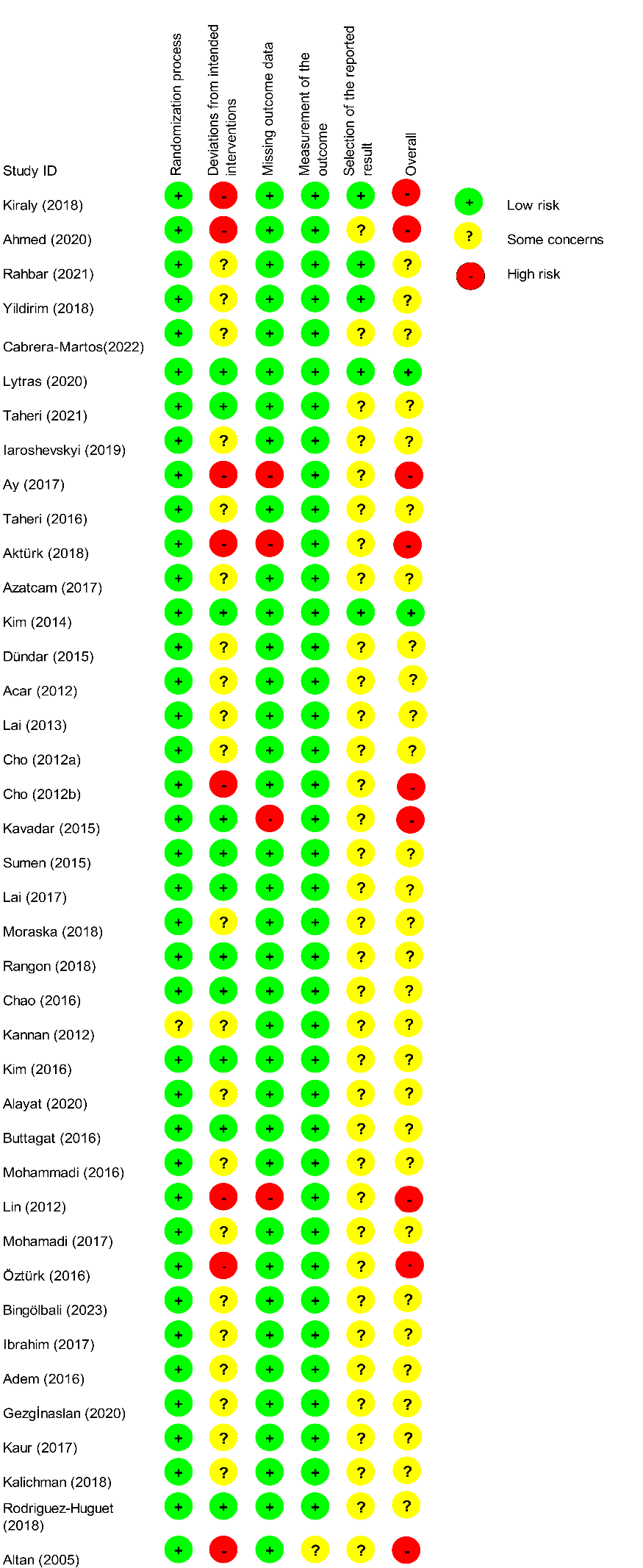
**Supplementary material 2.** Risk of bias for the included studies

**Supplementary material 3.** Risk of bias assessed with risk of bias tool for included studies


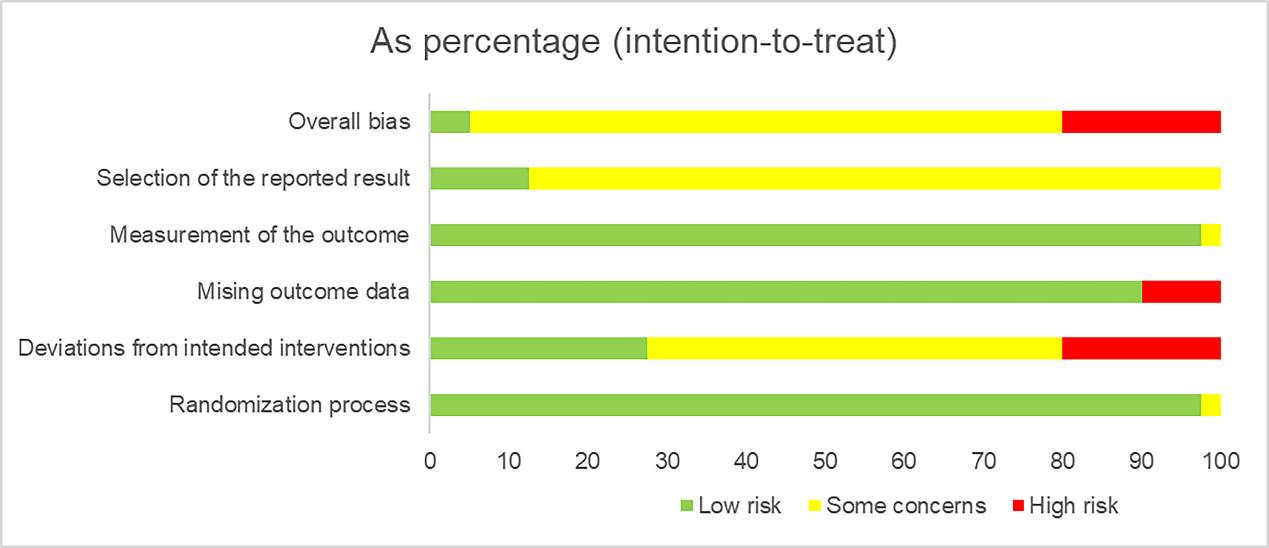


**Supplementary material 4.1.** Summary table of all data included in the network for pain intensity

| Author  (year) | Treatment arm 1 | | | Treatment arm 2 | | | Treatment arm 3 | | | Treatment arm 4 | | |
| --- | --- | --- | --- | --- | --- | --- | --- | --- | --- | --- | --- | --- |
|  | Treat^*^ | Mean ± SD | n | Treat^*^ | Mean ± SD | n | Treat^*^ | Mean ± SD | n | Treat^*^ | Mean ± SD | n |
| Kiraly  (2018) | 3 | 2.52 ± 1.86 | 31 | 5 | 2.57 ± 2.56 | 30 | NA | NA | NA | NA | NA | NA |
| Ahmed (2020) | 1 | 6.40 ± 0.90 | 15 | 2 | 3.50 ± 1.10 | 15 | 3 | 5.70 ± 0.90 | 15 | NA | NA | NA |
| Rahbar (2021) | 1 | 6.91 ± 2.03 | 23 | 5 | 5.72 ± 2.20 | 22 | 6 | 4.95 ± 2.86 | 24 | NA | NA | NA |
| Yildirim (2018) | 1 | 3.70 ± 1.80 | 27 | 6 | 1.90 ± 1.20 | 27 | NA | NA | NA | NA | NA | NA |
| Cabrera-Martos (2022) | 1 | 6.00 ± 2.00 | 20 | 2 | 4.00 ± 2.25 | 20 | NA | NA | NA | NA | NA | NA |
| Lytras  (2020) | 1 | 4.34 ± 1.00 | 20 | 2 | 3.33 ± 0.94 | 20 | NA | NA | NA | NA | NA | NA |
| Taheri (2021) | 5 | 5.50 ± 1.20 | 18 | 6 | 5.90 ± 2.30 | 19 | NA | NA | NA | NA | NA | NA |
| Iaroshevskyi (2019) | 1 | 4.29 ± 0.57 | 44 | 2 | 4.31 ± 0.68 | 43 | NA | NA | NA | NA | NA | NA |
| Ay  (2017) | 1 | 3.93 ± 1.96 | 30 | 9 | 2.35 ± 1.99 | 31 | NA | NA | NA | NA | NA | NA |
| Taheri (2016) | 3 | 4.20 ± 2.70 | 20 | 5 | 6.10 ± 2.40 | 26 | NA | NA | NA | NA | NA | NA |
| Aktürk (2018) | 1 | 6.71 ± 1.23 | 20 | 5 | 4.76 ± 1.98 | 20 | 6 | 6.24 ± 1.13 | 20 | NA | NA | NA |
| Azatcam (2017) | 1 | 4.78 ± 1.04 | 23 | 4 | 4.00 ± 0.90 | 23 | 9 | 3.00 ± 0.95 | 23 | NA | NA | NA |
| Kim  (2014) | 1 | 5.00 ± 1.80 | 25 | 4 | 4.54 ± 1.44 | 24 | 8 | 4.28 ± 1.65 | 25 | 10 | 4.16 ± 1.60 | 25 |
| Dündar (2015) | 1 | 4.20 ± 1.60 | 37 | 3 | 2.70 ± 1.20 | 38 | NA | NA | NA | NA | NA | NA |
| Acar  (2012) | 1 | 5.07 ± 2.18 | 20 | 7 | 3.72 ± 2.73 | 20 | 12 | 2.55 ± 1.76 | 20 | NA | NA | NA |
| Lai  (2014) | 1 | 4.38 ± 2.16 | 24 | 11 | 4.17 ± 1.45 | 24 | NA | NA | NA | NA | NA | NA |
| Cho  (2012) | 5 | 4.88 ± 1.36 | 12 | 7 | 5.42 ± 0.79 | 12 | 12 | 5.00 ± 1.53 | 12 | NA | NA | NA |
| Cho  (2012) | 1 | 4.34 ± 2.17 | 27 | 8 | 3.86 ± 1.64 | 30 | NA | NA | NA | NA | NA | NA |
| Kavadar (2015) | 1 | 2.28 ± 1.39 | 29 | 6 | 0.37 ± 0.89 | 30 | NA | NA | NA | NA | NA | NA |
| Sumen (2015) | 1 | 5.00 ± 1.77 | 15 | 3 | 3.80 ± 1.74 | 15 | 4 | 3.40 ± 1.50 | 15 | NA | NA | NA |
| Lai  (2017) | 1 | 4.37 ± 1.80 | 91 | 11 | 4.28 ± 1.82 | 98 | NA | NA | NA | NA | NA | NA |
| Moraska (2018) | 2 | 2.00 ± 1.40 | 12 | 6 | 1.70 ± 1.70 | 13 | NA | NA | NA | NA | NA | NA |
| Rangon (2018) | 1 | 6.00 ± 2.16 | 10 | 2 | 4.40 ± 2.87 | 10 | NA | NA | NA | NA | NA | NA |
| Chao  (2016) | 1 | 2.13 ± 1.64 | 15 | 9 | 3.56 ± 0.81 | 16 | NA | NA | NA | NA | NA | NA |
| Kannan (2012) | 2 | 2.02 ± 0.88 | 15 | 3 | 2.66 ± 1.23 | 15 | 6 | 2.34 ± 0.45 | 15 | NA | NA | NA |
| Kim  (2016) | 2 | 5.52 ± 1.26 | 23 | 6 | 4.45 ± 1.46 | 22 | NA | NA | NA | NA | NA | NA |
| Alayat (2020) | 1 | 3.60 ± 0.67 | 25 | 3 | 1.43 ± 0.77 | 25 | NA | NA | NA | NA | NA | NA |
| Mohammadi  (2016) | 1 | 5.88 ± 0.85 | 14 | 2 | 5.01 ± 0.73 | 14 | NA | NA | NA | NA | NA | NA |
| Lin  (2012) | 1 | 1.31 ± 0.78 | 26 | 8 | 1.12 ± 0.74 | 29 | NA | NA | NA | NA | NA | NA |
| Ozturk (2016) | 1 | 3.86 ± 2.60 | 20 | 9 | 3.05 ± 2.58 | 17 | NA | NA | NA | NA | NA | NA |
| Bingolbali (2023) | 1 | 4.70 ± 2.10 | 40 | 2 | 1.50 ± 1.20 | 40 | NA | NA | NA | NA | NA | NA |
| Rodriguez-Huguet (2017) | 2 | 2.35 ± 1.09 | 20 | 12 | 2.81 ± 1.33 | 21 | NA | NA | NA | NA | NA | NA |
| Yildirim (2016 | 1 | 5.10 ± 2.00 | 30 | 2 | 3.70 ± 2.40 | 30 | NA | NA | NA | NA | NA | NA |
| Kaur  (2017) | 2 | 3.50 ± 1.35 | 10 | 6 | 4.10 ± 0.99 | 10 | NA | NA | NA | NA | NA | NA |
| Gezgİnaslan (2020) | 5 | 3.70 ± 1.70 | 49 | 12 | 6.50 ± 1.20 | 45 | NA | NA | NA | NA | NA | NA |
| Buttagat (2016) | 1 | 3.84 ± 2.65 | 25 | 2 | 2.77 ± 1.94 | 25 | NA | NA | NA | NA | NA | NA |
| Altan  (2005) | 1 | 3.92 ± 0.42 | 25 | 3 | 4.13 ± 0.58 | 23 | NA | NA | NA | NA | NA | NA |
| ^*^Treatment types: 1 control or placebo, 2 manual therapy, 3 laser therapy, 4 electronic stimulation therapy, 5 extracorporeal shock wave therapy, 6 ultrasound, 7 exercise, 8 medicine, 9 Kinesio taping, 10 heat pack, 11 far-infrared ray, 12 combination therapy. NA, not applicable; SD, standard deviation. | | | | | | | | | | | | |

**Supplementary material 4.2.** Summary table of all data included in the network for pressure pain threshold

| Author  (year) | Treatment 1 | | | Treatment 2 | | | Treatment 3 | | | Treatment 4 | | |
| --- | --- | --- | --- | --- | --- | --- | --- | --- | --- | --- | --- | --- |
|  | Treat^*^ | Mean ± SD | n | Treat^*^ | Mean ± SD | n | Treat^*^ | Mean ± SD | n | Treat^*^ | Mean ± SD | n |
| Ahmed (2020) | 1 | 1.70 ± 0.40 | 15 | 2 | 2.20 ± 0.50 | 15 | 3 | 2.40 ± 0.40 | 15 | NA | NA | NA |
| Rahbar (2021) | 1 | 1.97 ± 0.59 | 23 | 5 | 2.29 ± 0.56 | 22 | 6 | 1.98 ± 0.52 | 24 | NA | NA | NA |
| Yildirim (2018) | 1 | 5.40 ± 0.70 | 27 | 6 | 7.40 ± 1.10 | 27 | NA | NA | NA | NA | NA | NA |
| Lytras (2020) | 1 | 3.04 ± 0.42 | 20 | 2 | 3.50 ± 0.52 | 20 | NA | NA | NA | NA | NA | NA |
| Ay  (2017) | 1 | 7.14 ± 1.03 | 30 | 9 | 7.81 ± 0.72 | 31 | NA | NA | NA | NA | NA | NA |
| Aktürk (2018) | 1 | 2.30 ± 0.80 | 20 | 5 | 3.44 ± 0.89 | 20 | 6 | 3.20 ± 0.71 | 20 | NA | NA | NA |
| Azatcam (2017) | 1 | 2.05 ± 0.31 | 23 | 4 | 2.12 ± 0.30 | 23 | 9 | 2.02 ± 0.26 | 23 | NA | NA | NA |
| Kim  (2014) | 1 | 2.47 ± 0.83 | 25 | 4 | 2.79 ± 1.41 | 24 | 8 | 2.59 ± 0.89 | 25 | 10 | 2.67 ± 0.74 | 25 |
| Lai  (2014) | 1 | 2.42 ± 0.66 | 24 | 11 | 2.47 ± 0.82 | 24 | NA | NA | NA | NA | NA | NA |
| Cho  (2012) | 5 | 36.26 ± 8.83 | 12 | 7 | 40.82 ± 9.43 | 12 | 12 | 33.30 ± 9.46 | 12 | NA | NA | NA |
| Sumen (2015) | 1 | 26.66 ± 8.37 | 15 | 3 | 30.06 ± 7.18 | 15 | 4 | 29.73 ± 8.85 | 15 | NA | NA | NA |
| Lai  (2017) | 1 | 2.87 ± 1.60 | 91 | 11 | 2.90 ± 1.53 | 98 | NA | NA | NA | NA | NA | NA |
| Moraska (2018) | 2 | 16.40 ± 5.80 | 12 | 6 | 11.90 ± 6.60 | 13 | NA | NA | NA | NA | NA | NA |
| Rangon (2018) | 1 | 1.62 ± 0.74 | 10 | 2 | 1.80 ± 0.74 | 10 | NA | NA | NA | NA | NA | NA |
| Chao  (2016) | 1 | 3.48 ± 0.38 | 15 | 9 | 3.68 ± 0.46 | 16 | NA | NA | NA | NA | NA | NA |
| Kim  (2016) | 2 | 2.05 ± 0.42 | 23 | 6 | 2.06 ± 0.84 | 22 | NA | NA | NA | NA | NA | NA |
| Alayat (2020) | 1 | 1.90 ± 0.32 | 25 | 3 | 2.85 ± 0.49 | 25 | NA | NA | NA | NA | NA | NA |
| Mohammadi (2016) | 1 | 1.51 ± 0.12 | 14 | 2 | 1.62 ± 0.10 | 14 | NA | NA | NA | NA | NA | NA |
| Lin  (2012) | 1 | 2.27 ± 0.97 | 26 | 8 | 2.65 ± 1.35 | 29 | NA | NA | NA | NA | NA | NA |
| Mohamadi (2017) | 2 | 2.10 ± 0.11 | 29 | 9 | 1.87 ± 0.11 | 29 | NA | NA | NA | NA | NA | NA |
| Ozturk (2016) | 1 | 5.93 ± 2.87 | 17 | 9 | 6.00 ± 3.61 | 20 | NA | NA | NA | NA | NA | NA |
| Rodriguez-Huguet (2017) | 2 | 2.52 ± 0.77 | 20 | 12 | 2.03 ± 0.57 | 21 | NA | NA | NA | NA | NA | NA |
| Ibrahim (2017) | 2 | 1.12 ± 0.32 | 15 | 5 | 1.33 ± 0.51 | 15 | NA | NA | NA | NA | NA | NA |
| Yildirim (2016) | 1 | 4.16 ± 2.23 | 30 | 2 | 4.89 ± 1.99 | 30 | NA | NA | NA | NA | NA | NA |
| Kalichman （2017） | 1 | 4.10 ± 1.67 | 25 | 9 | 6.03 ± 2.78 | 25 | NA | NA | NA | NA | NA | NA |
| Altan (2005） | 1 | 77.69 ± 2.69 | 25 | 3 | 80.26 ± 2.54 | 23 | NA | NA | NA | NA | NA | NA |
| ^*^Treatment types: 1 control or placebo, 2 manual therapy, 3 laser therapy, 4 electronic stimulation therapy, 5 extracorporeal shock wave therapy, 6 ultrasound, 7 exercise, 8 medicine, 9 Kinesio taping, 10 heat pack, 11 far-infrared ray, 12 combination therapy. NA, not applicable; SD, standard deviation. | | | | | | | | | | | | |

**Supplementary material 4.3.** Summary table of all data included in the network for pain-related disability

| Author  (year) | Treatment 1 | | | Treatment 2 | | | Treatment 3 | | | Treatment 4 | | |
| --- | --- | --- | --- | --- | --- | --- | --- | --- | --- | --- | --- | --- |
|  | Treat^*^ | Mean ± SD | n | Treat* | Mean ± SD | n | Treat^*^ | Mean ± SD | n | Treat^*^ | Mean ± SD | n |
| Kiraly  (2018) | 3 | 10.51 ± 7.28 | 31 | 5 | 10.38 ± 6.90 | 30 | NA | NA | NA | NA | NA | NA |
| Ahmed (2020) | 1 | 17.20 ± 6.10 | 15 | 2 | 13.30 ± 3.40 | 15 | 3 | 14.20 ± 5.40 | 15 | NA | NA | NA |
| Rahbar (2021) | 1 | 41.76 ± 16.81 | 23 | 5 | 39.04 ± 19.58 | 22 | 6 | 32.10 ± 18.34 | 24 | NA | NA | NA |
| Lytras (2020) | 1 | 23.35 ± 7.27 | 20 | 2 | 19.16 ± 4.93 | 20 | NA | NA | NA | NA | NA | NA |
| Taheri (2021) | 5 | 22.80 ± 5.50 | 18 | 6 | 24.1 ± 6.40 | 19 | NA | NA | NA | NA | NA | NA |
| Ay  (2017) | 1 | 36.10 ± 12.16 | 30 | 9 | 35.67 ± 20.27 | 31 | NA | NA | NA | NA | NA | NA |
| Taheri (2016) | 3 | 22.90 ± 21.40 | 20 | 5 | 42.70 ± 20.40 | 26 | NA | NA | NA | NA | NA | NA |
| Azatcam (2017) | 1 | 10.95 ± 4.52 | 23 | 4 | 10.08 ± 2.79 | 23 | 9 | 9.52 ± 3.71 | 23 | NA | NA | NA |
| Kim  (2014) | 1 | 10.72 ± 10.72 | 25 | 4 | 9.67 ± 2.82 | 24 | 8 | 10.16 ± 5.37 | 25 | 10 | 11.92 ± 4.90 | 25 |
| Kim  (2016) | 1 | 32.90 ± 8.30 | 37 | 3 | 32.60 ± 6.60 | 38 | NA | NA | NA | NA | NA | NA |
| Cho  (2012) | 5 | 22.33 ± 5.56 | 12 | 7 | 22.25 ± 4.88 | 12 | 12 | 22.58 ± 4.79 | 12 | NA | NA | NA |
| Cho  (2012) | 1 | 20.04 ± 13.17 | 27 | 8 | 17.47 ± 9.31 | 30 | NA | NA | NA | NA | NA | NA |
| Sumen (2015) | 1 | 28.40 ± 11.01 | 15 | 3 | 23.66 ± 9.32 | 15 | 4 | 26.86 ± 13.33 | 15 | NA | NA | NA |
| Lin  (2012) | 1 | 6.78 ± 3.59 | 26 | 8 | 5.15 ± 3.84 | 29 | NA | NA | NA | NA | NA | NA |
| Bingölbali (2023) | 1 | 66.30 ± 12.20 | 40 | 2 | 56.90 ± 9.10 | 40 | NA | NA | NA | NA | NA | NA |
| Ibrahim (2017) | 2 | 24.50 ± 9.31 | 15 | 5 | 22.74 ± 7.62 | 15 | NA | NA | NA | NA | NA | NA |
| Yildirim (2016) | 1 | 13.90 ± 6.50 | 30 | 2 | 11.00 ± 6.20 | 30 | NA | NA | NA | NA | NA | NA |
| Kaur  (2017) | 2 | 29.30 ± 6.15 | 10 | 6 | 35.00 ± 10.54 | 10 | NA | NA | NA | NA | NA | NA |
| Gezgİnaslan (2020) | 5 | 22.20 ± 7.80 | 49 | 12 | 33.50 ± 6.60 | 45 | NA | NA | NA | NA | NA | NA |
| ^*^Treatment types: 1 control or placebo, 2 manual therapy, 3 laser therapy, 4 electronic stimulation therapy, 5 extracorporeal shock wave therapy, 6 ultrasound, 7 exercise, 8 medicine, 9 Kinesio taping, 10 heat pack, 11 far-infrared ray, 12 combination therapy. NA, not applicable; SD, standard deviation. | | | | | | | | | | | | |

**Supplementary material 5.** Similarity comparison of treatment. (A) age by direct treatment comparisons; (B) percentage female by direct treatment comparisons; (C) baseline pain intensity by direct treatment comparisons; (D) baseline pressure pain threshold by direct treatment comparisons; (E) baseline pain-related disability by direct treatment comparisons. CN, control; CT, combination therapy; ENS, electrical nerve stimulation; ESWT, extracorporeal shock wave therapy; EX, exercise; FIR, far-infrared ray; HT, heat pack; KT, Kinesio taping; LT, laser therapy; ME, medication; MT, manual therapy; US, ultrasound.


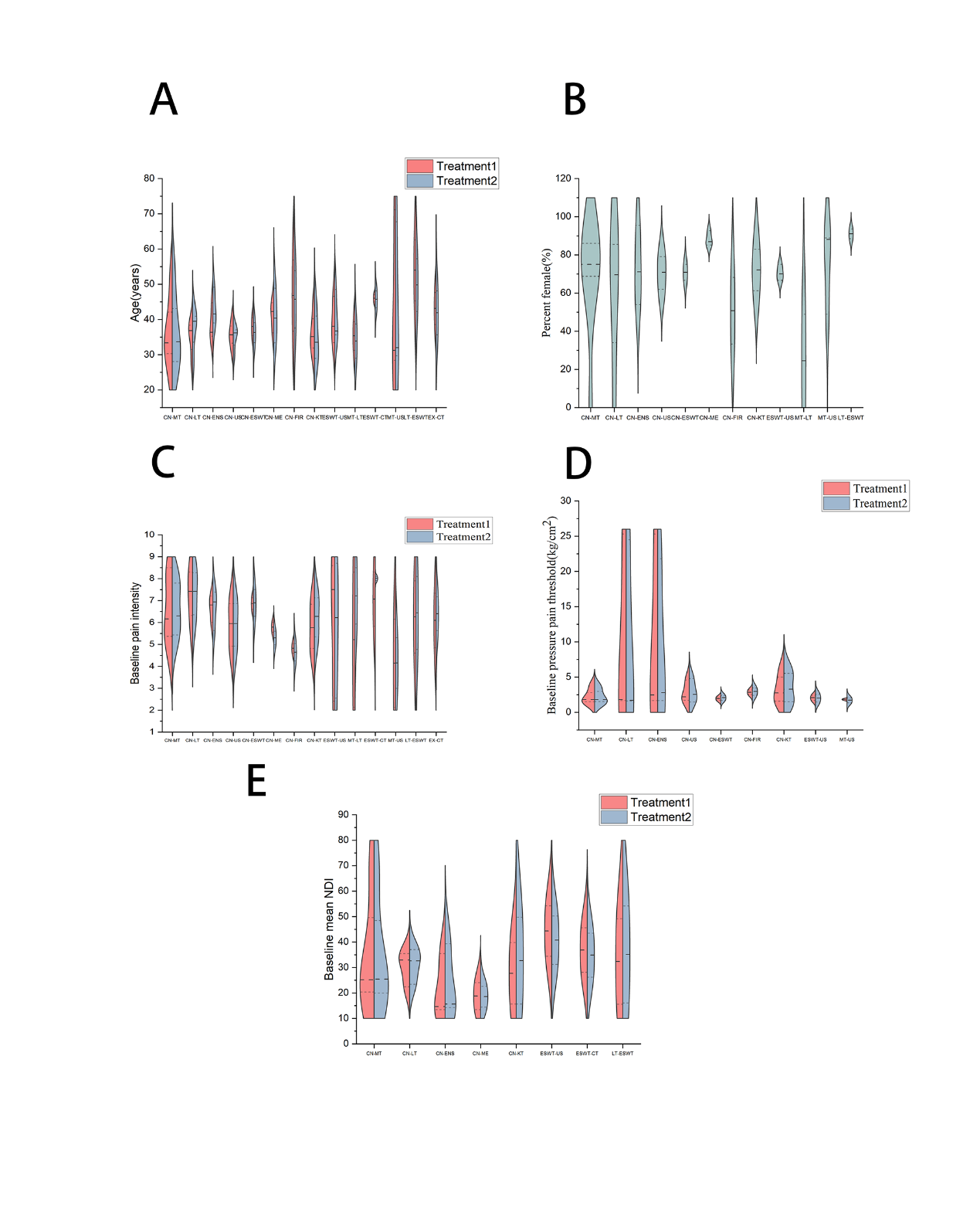


**Supplementary material 6.** Pairwise comparisons of meta-analysis of pain intensity, pressure pain threshold and pain-related disability

| **Comparison** | | **Author (year)** | **Pain intensity** | | | **Pressure pain threshold** | | | **Pain-related disability** | | |
| --- | --- | --- | --- | --- | --- | --- | --- | --- | --- | --- | --- |
|  |  |  | **MD** | **95% CI** | ***τ*^2^** | **MD** | **95% CI** | ***τ*^2^** | **MD** | **95% CI** | ***τ*^2^** |
| MT | Control | Ahmed (2020) | -2.90 | -3.62, -2.18 |  | 0.50 | 0.18, 0.82 |  | -3.90 | -7.43, -0.37 |  |
|  |  | Cabrera-Martos (2022) | -2.00 | -3.32, -0.68 |  | NR | NR |  | NR | NR |  |
|  |  | Lytras (2020) | -1.01 | -1.61, -0.41 |  | 0.46 | 0.17, 0.75 |  | -4.19 | -8.04, -0.34 |  |
|  |  | Iaroshevskyi (2019) | 0.02 | -0.24, 0.28 |  | NR | NR |  | NR | NR |  |
|  |  | Yildirim (2016) | -1.40 | -2.52, -0.28 |  | 0.73 | -0.34, 1.80 |  | -2.90 | -6.11, 0.31 |  |
|  |  | Rangon (2018) | -1.60 | -3.83, 0.63 |  | 0.18 | -0.47, 0.83 |  | NR | NR |  |
|  |  | Buttagat (2016) | -1.07 | -2.36, 0.22 |  | NR | NR |  | NR | NR |  |
|  |  | Mohammadi (2016) | -0.87 | -1.46, -0.28 |  | 0.11 | 0.03, 0.19 |  | NR | NR |  |
|  |  | Bingölbali (2023) | -3.20 | -3.95, -2.45 |  | NR | NR |  | -9.40 | -14.12, -4.68 |  |
|  |  | **Summary effect** | -1.54 | -2.44, -0.64 |  | 0.32 | 0.08, 0.57 |  | -4.71 | -7.20, -2.22 |  |
|  |  | **Heterogeneity (*I*^2^)** | 93% |  | 1.63 | 63% |  | 0.04 | 43% |  | 2.74 |
| LT | Control | Ahmed (2020) | -0.70 | -1.34, -0.06 |  | 0.70 | 0.41, 0.99 |  | -3.00 | -7.12, 1.12 |  |
|  |  | Dündar (2015) | -1.50 | -2.14, -0.86 |  | NR | NR |  | -0.30 | -3.70, 3.10 |  |
|  |  | Sumen (2015) | -1.20 | -2.46, 0.06 |  | 3.40 | -2.18, 8.98 |  | -4.74 | -1.18, 0.27 |  |
|  |  | Alayat (2020) | -2.17 | -2.57, -1.77 |  | 0.95 | 0.72, 1.18 |  | NR | NR |  |
|  |  | Altan (2005) | 0.21 | -0.08, 0.50 |  | 2.57 | 1.09, 4.05 |  | NR | NR |  |
|  |  | **Summary effect** | -1.06 | -2.20, 0.07 |  | 0.96 | 0.55, 1.37 |  | -1.78 | -4.24, 0.69 |  |
|  |  | **Heterogeneity (*I*^2^)** | 96% |  | 1.56 | 61% |  | 0.08 | 0% |  | 0 |
| ENS | Control | Azatcam (2017) | -0.78 | -1.34, -0.22 |  | 0.07 | -0.11, 0.24 |  | -0.87 | -3.04, 1.30 |  |
|  |  | Kim (2014) | -0.46 | -1.37, 0.45 |  | 0.32 | -0.33, 0.97 |  | -1.05 | -3.13, 1.03 |  |
|  |  | Sumen (2015) | -1.60 | -2.77, -0.43 |  | 33.07 | -3.09, 9.23 |  | -1.54 | -10.29, 7.21 |  |
|  |  | **Summary effect** | -0.83 | -1.33, -0.34 |  | 0.08 | -0.08, 0.25 |  | -0.22 | -0.57, 0.13 |  |
|  |  | **Heterogeneity (*I*^2^)** | 14% |  | 0.03 | 0% |  | 0.08 | 0% |  | 0 |
| ESWT | Control | Ibrahim (2017) | -1.19 | -2.43, 0.05 |  | 0.32 | -0.02, 0.66 |  | -2.72 | -13.40, 77.96 |  |
|  |  | Aktürk (2018) | -1.95 | -2.97, -0.93 |  | 1.14 | 0.62, 1.66 |  | NR | NR |  |
|  |  | Gezgİnaslan (2020) | -2.80 | -3.39, -2.21 |  | NR | NR |  | -11.30 | -14.21, -8.39 |  |
|  |  | **Summary effect** | -2.10 | -3.04, -1.16 |  | 0.70 | -0.10, 1.51 |  | -8.61 | -16.41, -0.81 |  |
|  |  | **Heterogeneity (*I*^2^)** | 68% |  | 0.46 | 85% |  | 0.29 | 57% |  | 20.85 |
| US | Control | Rahbar (2021) | -1.96 | -3.37, -0.55 |  | 0.01 | -0.31, 0.33 |  | -9.66 | -19.71, 0.39 |  |
|  |  | Yildirim (2018) | -1.80 | -2.62, -0.98 |  | 2.00 | 1.51, 2.49 |  | NR | NR |  |
|  |  | Aktürk (2018) | -0.47 | -1.20, 0.26 |  | 0.90 | 0.43, 1.37 |  | NR | NR |  |
|  |  | Kavadar (2015) | -1.91 | -2.51, -1.31 |  | NR | NR |  | NR | NR |  |
|  |  | **Summary effect** | -1.49 | -2.26, -0.73 |  | 0.96 | -0.20, 2.12 |  | -9.66 | -19.71, 0.39 |  |
|  |  | **Heterogeneity (*I*^2^)** | 71% |  | 0.42 | 96% |  | 1.01 | NA |  | NA |
| EX | Control | Acar (2012) | -1.35 | -2.88, 0.18 |  | NR | NR |  | NR | NR |  |
|  |  | **Summary effect** | -1.35 | -2.88, 0.18 |  | NA | NA |  | NA | NA |  |
|  |  | **Heterogeneity (*I*^2^)** | NA |  | NA | NA |  | NA | NA |  | NA |
| ME | Control | Kim (2014) | -0.72 | -1.68, 0.24 |  | 0.12 | -0.36, 0.60 |  | -0.56 | -3.30, 2.18 |  |
|  |  | Cho (2012) | -0.48 | -1.49, 0.53 |  | NR | NR |  | -2.57 | -8.55, 3.41 |  |
|  |  | Lin (2012) | -0.19 | -0.59, 0.21 |  | 0.38 | -0.24, 1.00 |  | -1.63 | -3.59, 0.33 |  |
|  |  | **Summary effect** | -0.29 | -0.64, 0.05 |  | 0.22 | -0.16, 0.59 |  | -1.35 | -2.89, 0.19 |  |
|  |  | **Heterogeneity (*I*^2^)** | 0% |  | 0 | 0% |  | 0 | 0% |  | 0 |
| KT | Control | Ay (2017) | -1.58 | -2.57, -0.59 |  | 0.67 | 0.22, 1.11 |  | -0.43 | -8.79, 7.93 |  |
|  |  | Azatcam (2017) | -1.78 | -2.36, -1.20 |  | -0.03 | -0.19, 0.14 |  | -1.43 | -3.82, 0.96 |  |
|  |  | Chao (2016) | 1.43 | 0.51, 2.35 |  | 0.20 | -0.10, 0.49 |  | NR | NR |  |
|  |  | Ozturk (2016) | 0.81 | -0.86, 2.48 |  | 0.07 | -2.02, 2.16 |  | NR | NR |  |
|  |  | Kalichman (2018) | NR | NR |  | 1.93 | 0.29, 3.57 |  | NR | NR |  |
|  |  | **Summary effect** | -0.34 | -2.04, 1.37 |  | 0.31 | -0.07, 0.69 |  | -1.35 | -3.65, 0.94 |  |
|  |  | **Heterogeneity (*I*^2^)** | 92% |  | 2.72 | 71% |  | 0.10 | 0% |  | 0 |
| HT | Control | Kim (2014) | -0.84 | -1.78, 0.10 |  | 0.20 | -0.24, 0.64 |  | 1.20 | -1.40, 3.80 |  |
|  |  | **Summary effect** | -0.84 | -1.78, 0.10 |  | 0.20 | -0.24, 0.64 |  | 1.20 | -1.40, 3.80 |  |
|  |  | **Heterogeneity (*I*^2^)** | NA |  | NA | NA |  | NA | NA |  | NA |
| FIR | Control | Lai (2014) | -0.21 | -1.25, 0.83 |  | 0.05 | -0.37, 0.47 |  | NR | NR |  |
|  |  | Lai (2017) | -0.09 | -0.61, 0.43 |  | 0.03 | -0.42, 0.48 |  | NR | NR |  |
|  |  | **Summary effect** | -0.11 | -0.58, 0.35 |  | 0.04 | -0.27, 0.35 |  | NA | NA |  |
|  |  | **Heterogeneity (*I*^2^)** | 0% |  | 0 | 0% |  | 0 | NA |  | NA |
| CT | Control | Acar (2012) | -2.52 | -3.75, -1.29 |  | NR | NR |  | NR | NR |  |
|  |  | **Summary effect** | -2.52 | -3.75, -1.29 |  | NA | NA |  | NA | NA |  |
|  |  | **Heterogeneity (*I*^2^)** | NA |  | NA | NA |  | NA | NA |  | NA |
| MT | LT | Kannan (2012) | -0.64 | -1.41, 0.13 |  | NR | NR |  | NR | NR |  |
|  |  | Ahmed (2020) | -2.20 | -2.92, -1.48 |  | -0.20 | -0.52, 0.12 |  | -0.90 | -4.13, 2.33 |  |
|  |  | **Summary effect** | -1.43 | -2.95, 0.10 |  | -0.20 | -0.52, 0.12 |  | -0.90 | -4.13, 2.33 |  |
|  |  | **Heterogeneity (*I*^2^)** | 88% |  | 1.07 | NA |  | NA | NA |  | NA |
| MT | KT | Mohamadi (2017) | NR | NR |  | 2.06 | 1.42, 2.71 |  | NR | NR |  |
|  |  | **Summary effect** | NA | NA |  | 2.06 | 1.42, 2.71 |  | NA | NA |  |
|  |  | **Heterogeneity (*I*^2^)** | NA |  | NA | NA |  | NA | NA |  | NA |
| MT | ESWT | Ibrahim (2017) | NR | NR |  | -0.21 | -0.52, 0.09 |  | 1.76 | -4.33, 7.85 |  |
|  |  | **Summary effect** | NA | NA |  | -0.21 | -0.52, 0.09 |  | 1.76 | -4.33, 7.85 |  |
|  |  | **Heterogeneity (*I*^2^)** | NA |  | NA | NA |  | NA | NA |  | NA |
| MT | US | Moraska (2018) | 0.30 | -0.92, 1.52 |  | 4.50 | -0.36, 99.36 |  | NR | NR |  |
|  |  | Kannan (2012) | -0.32 | -0.82, 0.18 |  | NR | NR |  | NR | NR |  |
|  |  | Kim (2016) | 1.07 | 0.27, 1.87 |  | -0.01 | -0.40, 0.338 |  | NR | NR |  |
|  |  | Kaur (2017) | -0.60 | -1.64, 0.44 |  | NR | NR |  | -5.70 | -13.26, 1.86 |  |
|  |  | **Summary effect** | 0.11 | -0.66, 0.87 |  | 1.57 | -2.65, 5.78 |  | -5.70 | -13.26, 1.86 |  |
|  |  | **Heterogeneity (*I*^2^)** | 70% |  | 0.41 | 70% |  | 7.07 | NA |  | NA |
| MT | CT | Rodriguez-Huguet (2018) | -0.37 | -0.99, 0.25 |  | 0.49 | 0.07, 0.91 |  | NR | NR |  |
|  |  | **Summary effect** | -0.37 | -0.99, 0.25 |  | 0.49 | 0.07, 0.91 |  | NA | NA |  |
|  |  | **Heterogeneity (*I*^2^)** | NA |  | NA | NA |  | NA | NA |  | NA |
| LT | ENS | Sumen (2015) | 0.40 | -0.76, 1.56 |  | 0.33 | -5.44, 6.10 |  | -3.20 | -11.43, 5.03 |  |
|  |  | **Summary effect** | 0.40 | -0.76, 1.56 |  | 0.33 | -5.44, 6.10 |  | -3.20 | -11.43, 5.03 |  |
|  |  | **Heterogeneity (*I*^2^)** | NA |  | NA | NA |  | NA | NA |  | NA |
| LT | ESWT | Kiraly (2018) | -0.05 | -1.18, 1.08 |  | NR | NR |  | 0.13 | -3.43, 3.69 |  |
|  |  | Taheri (2016) | -1.90 | -3.40, -0.40 |  | NR | NR |  | -19.80 | -32.02, -7.58 |  |
|  |  | **Summary effect** | -0.91 | -2.71, 0.90 |  | NA | NA |  | -8.94 | -28.39, 10.51 |  |
|  |  | **Heterogeneity (*I*^2^)** | 73% |  | 1.25 | NA |  | NA | 89% |  | 177.50 |
| LT | US | Kannan (2012) | 0.32 | -0.34, 0.98 |  | NR | NR |  | NR | NR |  |
|  |  | **Summary effect** | 0.32 | -0.34, 0.98 |  | NA | NA |  | NA | NA |  |
|  |  | **Heterogeneity (*I*^2^)** | NA |  | NA | NA |  | NA | NA |  | NA |
| ENS | ME | Kim (2014) | 0.26 | -0.61,1.13 |  | 0.20 | -0.46, 0.86 |  | -0.49 | -2.88, 1,90 |  |
|  |  | **Summary effect** | 0.26 | -0.61,1.13 |  | 0.20 | -0.46, 0.86 |  | -0.49 | -2.88, 1,90 |  |
|  |  | **Heterogeneity (*I*^2^)** | NA |  | NA | NA |  | NA | NA |  | NA |
| ENS | KT | Azatcam (2016) | 1.00 | 0.47, 1.53 |  | 0.09 | -0.07, 0.25 |  | 0.56 | -1.34, 2.46 |  |
|  |  | **Summary effect** | 1.00 | 0.47, 1.53 |  | 0.09 | -0.07, 0.25 |  | 0.56 | -1.34, 2.46 |  |
|  |  | **Heterogeneity (*I*^2^)** | NA |  | NA | NA |  | NA | NA |  | NA |
| ENS | HT | Kim (2014) | 0.26 | -0.61, 1.13 |  | 0.12 | -0.51, 0.75 |  | -2.25 | -4.48, -0.02 |  |
|  |  | **Summary effect** | 0.26 | -0.61, 1.13 |  | 0.12 | -0.51, 0.75 |  | -2.25 | -4.48, -0.02 |  |
|  |  | **Heterogeneity (*I*^2^)** | NA |  | NA | NA |  | NA | NA |  | NA |
| ESWT | US | Taheri (2021) | -0.40 | -1.57, 0.77 |  | NR | NR |  | -1.30 | -5.14, 2.54 |  |
|  |  | Rahbar (2021) | 0.77 | -0.70, 2.24 |  | 0.31 | -0.00, 0.62 |  | 6.94 | -4.05, 17.93 |  |
|  |  | Aktürk (2018) | -1.48 | -2.48, -0.48 |  | 0.24 | -0.26, 0.74 |  | NR | NR |  |
|  |  | **Summary effect** | -0.46 | -1.70, 0.78 |  | 0.29 | 0.03, 0.56 |  | 1.14 | -6.23, 8.52 |  |
|  |  | **Heterogeneity (*I*^2^)** | 69% |  | 0.82 | 0% |  | 0 | 48% |  | 16.31 |
| ESWT | EX | Cho (2012) | -0.54 | -1.43, 0.35 |  | -4.56 | -11.87, 2.75 |  | 0.08 | -4.11, 4.27 |  |
|  |  | **Summary effect** | -0.54 | -1.43, 0.35 |  | -4.56 | -11.87, 2.75 |  | 0.08 | -4.11, 4.27 |  |
|  |  | **Heterogeneity (*I*^2^)** | NA |  | NA | NA |  | NA | NA |  | NA |
| ESWT | CT | Cho (2012) | -0.12 | -1.28, 1.04 |  | 2.96 | -4.36, 10.28 |  | -0.25 | -4.40, 3.90 |  |
|  |  | **Summary effect** | -0.12 | -1.28, 1.04 |  | 2.96 | -4.36, 10.28 |  | -0.25 | -4.40, 3.90 |  |
|  |  | **Heterogeneity (*I*^2^)** | NA |  | NA | NA |  | NA | NA |  | NA |
| EX | CT | Cho (2012) | 0.42 | -0.55, 1.39 |  | 7.52 | -0.04, 15.08 |  | -0.33 | -4.20, 3.54 |  |
|  |  | Acar (2012) | 1.17 | -0.25, 2.59 |  | NR | NR |  | NR | NR |  |
|  |  | **Summary effect** | 0.66 | -0.14, 1.46 |  | 7.52 | -0.04, 15.08 |  | -0.33 | -4.20, 3.54 |  |
|  |  | **Heterogeneity (*I*^2^)** | 0% |  | 0 | NA |  | NA | NA |  | NA |
| ME | HT | Kim (2014) | 0.12 | -0.78, 1.02 |  | -0.08 | -0.53, 0.37 |  | -1.76 | -4.61, 1.09 |  |
|  |  | **Summary effect** | 0.12 | -0.78, 1.02 |  | -0.08 | -0.53, 0.37 |  | -1.76 | -4.61, 1.09 |  |
|  |  | **Heterogeneity (*I*^2^)** | NA |  | NA | NA |  | NA | NA |  | NA |
| CI, confidence interval; CT, combined therapy; ENS, electrical nerve stimulation; ESWT, extracorporeal shock wave therapy; EX, exercise; FIR, far-infrared ray; HT, heat; KT, Kinesio taping; LT, laser therapy; ME, medications; MT, manual therapy; NA, not applicable; NR, not reported; MD, mean difference; US, ultrasound. | | | | | | | | | | | |

**Supplementary material 7.** League table reporting the comparative effects for all interventions for pain-related disability

|  | MT |  |  |  |  |  |  |  |  | |  |
| --- | --- | --- | --- | --- | --- | --- | --- | --- | --- | --- | --- |
| Pain-related disability | -0.76  (-4.42, 2.90) | LT |  |  |  |  |  |  |  | |  |
|  | -4.17  (-8.37, 0.02) | -3.41  (-7.78, 0.96) | ENS |  |  |  |  |  |  | |  |
|  | 0.44  (-3.60, 4.48) | 1.20  (-2.91, 5.32) | 4.61  (-0.22, 9.45) | ESWT |  |  |  |  |  | |  |
|  | -0.76  (-5.96, 4.45) | 0.00  (-5.57, 5.58) | 3.42  (-2.62, 9.45) | -1.20  (-5.93, 3.53) | US |  |  |  |  | |  |
|  | 0.52  (-6.98, 8.03) | 1.28  (-6.26, 8.83) | 4.69  (-3.27, 12.65) | 0.08  (-6.24, 6.40) | 1.28  (-6.62, 9.17) | EX |  |  |  | |  |
|  | -4.00  (-8.25, 0.25) | -3.24  (-7.76, 1.29) | 0.17  (-3.83, 4.17) | -4.44  (-9.36, 0.47) | -3.24  (-9.33, 2.84) | -4.52  (-12.53, 3.49) | ME |  |  | |  |
|  | -3.96  (-9.07, 1.16) | -3.20  (-8.53, 2.14) | 0.21  (-4.25, 4.67) | -4.40  (-10.01, 1.21) | -3.20  (-9.89, 3.49) | -4.48  (-12.93, 3.98) | -2.30  (-8.40, 3.79) | KT |  | |  |
|  | **-6.26**  **(-11.74, -0.79)** | -5.50  (-11.17, 0.17) | -2.09  (-6.96, 2.78) | **-6.70**  **(-12.70, -0.71)** | -5.51  (-12.49, 1.48) | -6.78  (-15.49, 1.93) | 4.15  (-4.29, 12.59) | -2.27  (-8.88, 4.33) | HT |  |  |
|  | 0.19  (-7.29, 7.68) | 0.95  (-6.58, 8.48) | 4.36  (-3.58, 12.30) | -0.25  (-6.55, 6.05) | 0.95  (-6.93, 8.83) | -0.33  (-6.45, 5.79) | -1.38  (-5.70, 2.94) | -5.49  (-13.42, 2.44) | 6.45  (-2.24, 15.15) | CT |  |
|  | **-5.34**  **(-8.09, -2.58)** | **-4.58**  **(-7.80, -1.36)** | -1.17  (-4.39, 2.05) | **-5.78**  **(-9.45, -2.12)** | -4.58  (-9.72, 0.56) | -5.86  (-13.17, 1.45) | -1.34  (-4.60, 1.92) | -1.32  (-5.97, 3.33) | 0.92  (-3.83, 5.67) | -5.53  (-12.82, 1.76) | CN |

Date is presented by mean difference with 95% confidence intervals. Bold denotes statistical significance at *P* < 0.05.

CN, control; CT, combination therapy; ENS, electrical nerve stimulation; ESWT, extracorporeal shock wave therapy; EX, exercise; HT, heat; KT, Kinesio taping; LT, laser therapy; ME, medication; MT, manual therapy; US, ultrasound.

**Supplementary material 8.1.** Node-splitting analysis of pain intensity

| Comparison | Direct | | Indirect | | Difference | | *P* value |
| --- | --- | --- | --- | --- | --- | --- | --- |
|  | Coef. | Std. Err. | Coef. | Std. Err. | Coef. | Std. Err. |  |
| AB | -1.52 | 0.34 | -1.81 | 0.55 | 0.29 | 0.65 | 0.654 |
| AC | -1.06 | 0.43 | -1.33 | 0.62 | 0.27 | 0.75 | 0.716 |
| AD | -0.92 | 0.58 | 0.32 | 1.21 | -1.24 | 1.35 | 0.357 |
| AE | -2.03 | 0.59 | -1.18 | 0.59 | -0.85 | 0.83 | 0.305 |
| AF | -1.49 | 0.51 | -1.59 | 0.52 | 0.10 | 0.72 | 0.892 |
| AG | -1.40 | 1.19 | -0.81 | 0.99 | -0.59 | 1.54 | 0.701 |
| AH | -0.44 | 0.58 | -1.52 | 2.16 | 1.08 | 2.24 | 0.631 |
| AI | -0.71 | 0.52 | -2.12 | 2.10 | 1.41 | 2.17 | 0.517 |
| AJ | -0.84 | 1.02 | -0.88 | 1.72 | 0.04 | 2.02 | 0.986 |
| AL | -2.52 | 1.09 | -1.24 | 0.77 | -1.28 | 1.34 | 0.338 |
| BC | 1.43 | 0.66 | -0.08 | 0.50 | 1.50 | 0.83 | 0.070 |
| BF | -0.11 | 0.51 | 0.27 | 0.56 | -0.38 | 0.76 | 0.618 |
| BL | 0.46 | 0.98 | -0.46 | 0.84 | 0.92 | 1.29 | 0.475 |
| CD | -0.42 | 1.07 | 0.86 | 0.72 | -1.27 | 1.29 | 0.325 |
| CE | 0.87 | 0.76 | -1.21 | 0.56 | 2.08 | 0.95 | 0.028 |
| CF | -0.31 | 0.97 | -0.42 | 0.52 | 0.11 | 1.10 | 0.917 |
| DH | -0.26 | 1.01 | 0.54 | 0.94 | -0.80 | 1.38 | 0.562 |
| DI | -1.00 | 0.92 | 0.72 | 0.89 | -1.71 | 1.28 | 0.182 |
| DJ | -0.38 | 1.00 | 0.70 | 2.01 | -1.08 | 2.24 | 0.631 |
| EF | 0.47 | 0.63 | -0.42 | 0.68 | 0.90 | 0.92 | 0.331 |
| EG | 0.53 | 1.01 | 0.59 | 1.18 | -0.06 | 1.56 | 0.967 |
| EL | 0.12 | 1.08 | -0.18 | 0.86 | 0.30 | 1.38 | 0.828 |
| GL | -0.78 | 0.77 | 1.06 | 2.47 | -1.84 | 2.58 | 0.474 |
| HJ | -0.12 | 1.02 | -1.22 | 2.07 | 1.10 | 2.31 | 0.634 |
| A, control or placebo; B, manual therapy; C, laser therapy; Coef., coefficient; D, electrical nerve stimulation; E, extracorporeal shock wave therapy; F, ultrasound therapy; G, exercise; H, medications; I, Kinesio taping; J, heat; K, far-infrared ray; L, combination therapy; Std. Err, standard error. | | | | | | | |

**Supplementary material 8.2**. Node-splitting analysis of pressure pain threshold

| Comparison | Direct | | Indirect | | Difference | | *P* value |
| --- | --- | --- | --- | --- | --- | --- | --- |
|  | Coef. | Std. Err. | Coef. | Std. Err. | Coef. | Std. Err. |  |
| AB | 0.35 | 0.21 | 0.84 | 0.30 | -0.49 | 0.36 | 0.179 |
| AC | 1.05 | 0.30 | 0.63 | 0.87 | 0.42 | 0.92 | 0.647 |
| AD | 0.20 | 0.33 | 0.98 | 0.72 | -0.78 | 0.78 | 0.317 |
| AE | 0.69 | 0.33 | 1.10 | 0.44 | -0.40 | 0.55 | 0.464 |
| AF | 0.91 | 0.27 | 0.32 | 0.47 | 0.59 | 0.54 | 0.274 |
| AH | 0.24 | 0.36 | 0.08 | 1.24 | 0.16 | 1.29 | 0.901 |
| AI | 0.41 | 0.25 | 0.33 | 0.44 | 0.08 | 0.50 | 0.868 |
| AJ | 0.20 | 0.48 | 0.41 | 0.97 | -0.21 | 1.09 | 0.848 |
| BC | 0.20 | 0.45 | 0.72 | 0.42 | -0.52 | 0.62 | 0.395 |
| BE | 0.22 | 0.45 | 0.37 | 0.35 | -0.16 | 0.57 | 0.782 |
| BF | -0.14 | 0.46 | 0.41 | 0.31 | -0.55 | 0.55 | 0.320 |
| BI | -0.23 | 0.43 | -0.09 | 0.31 | -0.14 | 0.53 | 0.793 |
| BL | -0.49 | 0.46 | -2.64 | 3.75 | 2.15 | 3.78 | 0.569 |
| CD | -0.66 | 2.95 | -0.68 | 0.40 | 0.02 | 2.98 | 0.995 |
| DH | -0.21 | 0.54 | 0.06 | 0.64 | -0.27 | 0.84 | 0.752 |
| DI | -0.09 | 0.43 | 0.29 | 0.52 | -0.38 | 0.67 | 0.571 |
| DJ | -0.12 | 0.53 | 0.04 | 1.12 | -0.16 | 1.29 | 0.901 |
| EF | -0.28 | 0.31 | 0.49 | 0.52 | -0.76 | 0.60 | 0.205 |
| EG | 4.56 | 3.75 | 8.89 | 6.74 | -4.33 | 7.59 | 0.568 |
| EL | -2.96 | 3.76 | -0.79 | 0.53 | -2.17 | 3.80 | 0.568 |
| GL | -7.52 | 3.88 | -3.19 | 6.52 | -4.33 | 7.59 | 0.568 |
| HJ | 0.08 | 0.48 | -0.45 | 1.29 | 0.53 | 1.39 | 0.702 |
| A, control or placebo; B, manual therapy; C, laser therapy; Coef., coefficient; D, electrical nerve stimulation; E, extracorporeal shock wave therapy; F, ultrasound therapy; G, exercise; H, medications; I, Kinesio taping; J, heat; K, far-infrared ray; L, combination therapy; Std. Err, standard error. | | | | | | | |

**Supplementary material 8.3.** Node-splitting analysis of pain-related disability

| Comparison | Direct | | Indirect | | Difference | | *P* value |
| --- | --- | --- | --- | --- | --- | --- | --- |
|  | Coef. | Std. Err. | Coef. | Std. Err. | Coef. | Std. Err. |  |
| AB | -4.83 | 1.56 | -7.74 | 3.42 | 2.90 | 3.79 | 0.444 |
| AC | -1.92 | 1.56 | -9.70 | 2.34 | 7.78 | 2.81 | 0.006 |
| AD | -1.04 | 1.88 | -2.09 | 4.89 | 1.04 | 5.24 | 0.842 |
| AE | -10.77 | 1.43 | -2.25 | 1.68 | -8.52 | 2.21 | 0.000 |
| AF | -9.49 | 5.71 | -3.21 | 3.07 | -6.28 | 6.48 | 0.333 |
| AH | -1.43 | 1.86 | -0.38 | 6.96 | -1.04 | 7.19 | 0.885 |
| AI | -1.17 | 2.56 | -2.65 | 6.81 | 1.48 | 7.30 | 0.839 |
| AJ | 1.20 | 3.02 | -0.04 | 5.42 | 1.24 | 6.17 | 0.841 |
| BC | 0.79 | 3.14 | 0.67 | 2.56 | 0.12 | 4.04 | 0.976 |
| BE | -1.76 | 4.09 | 0.22 | 2.57 | -1.98 | 4.83 | 0.681 |
| BF | 5.70 | 4.49 | -1.75 | 3.15 | 7.45 | 5.48 | 0.174 |
| CD | 3.24 | 4.87 | 3.51 | 2.64 | -0.27 | 5.54 | 0.961 |
| CE | 2.23 | 2.51 | -6.05 | 2.35 | 8.29 | 3.08 | 0.007 |
| DH | 0.46 | 2.93 | -1.00 | 3.34 | 1.47 | 4.45 | 0.741 |
| DI | -0.56 | 2.82 | 0.90 | 4.82 | -1.46 | 5.57 | 0.793 |
| DJ | 2.25 | 2.93 | 1.21 | 6.66 | 1.04 | 7.19 | 0.885 |
| EF | -0.39 | 2.75 | 6.01 | 4.72 | -6.40 | 5.42 | 0.238 |
| EG | -0.08 | 3.23 | 11.38 | 321.15 | -11.46 | 321.16 | 0.972 |
| EL | 0.25 | 3.22 | 11.71 | 321.10 | -11.46 | 321.12 | 0.972 |
| HJ | 1.76 | 3.04 | 4.59 | 6.55 | -2.83 | 7.24 | 0.696 |
| A, control or placebo; B, manual therapy; C, laser therapy; Coef., coefficient; D, electrical nerve stimulation; E, extracorporeal shock wave therapy; F, ultrasound therapy; G, exercise; H, medications; I, Kinesio taping; J, heat; L, combination therapy; Std. Err, standard error. | | | | | | | |

**Supplementary material 8.4.** Global Wald test for inconsistency models

|  | Wald *χ*^2^ | *P* value |
| --- | --- | --- |
| Pain intensity | 12.58 | 0.764 |
| Pressure pain threshold | 16.57 | 0.167 |
| Pain-related disability | 5.80 | 0.926 |

**Supplementary material 9.1.** GRADE assessment for all pairwise comparisons within the pain intensity network

| Comparison | Confidence | Downgrading due to |
| --- | --- | --- |
| Manual *vs.* Control | Low | Study limitation; inconsistency |
| Laser *vs.* Control | Low | Study limitation; inconsistency |
| ENS *vs.* Control | Moderate | Imprecision |
| ESWT *vs.* Control | Low | Study limitation; inconsistency |
| US *vs.* Control | Low | Study limitation; inconsistency |
| Exercise *vs.* Control | Low | Study limitation; imprecision |
| ME *vs.* Control | Low | Study limitation; imprecision |
| KT *vs.* Control | Low | Study limitation; imprecision |
| HT *vs.* Control | Moderate | Imprecision |
| FIR *vs.* Control | Low | Study limitation; imprecision |
| CT *vs.* Control | Low | Study limitation; imprecision |
| Manual *vs.* Laser | Very low | Study limitation; inconsistency; imprecision |
| Manual *vs.* US | Low | Study limitation; imprecision |
| Manual *vs.* CT | Moderate | Imprecision |
| Manual *vs.* FIR | Low | Study limitation; inconsistency |
| Laser *vs.* ENS | Low | Study limitation; imprecision |
| Laser *vs.* ESWT | Very low | Study limitation; inconsistency; imprecision |
| Laser *vs.* US | Low | Study limitation; imprecision |
| ENS *vs.* ME | Low | Study limitation; imprecision |
| ENS *vs.* KT | Low | Study limitation; imprecision |
| ENS *vs.* HT | Very low | Imprecision |
| ESWT *vs.* US | Low | Study limitation; imprecision |
| ESWT *vs.* EX | Low | Study limitation; imprecision |
| ESWT *vs.* CT | Very low | Study limitation; imprecision |
| EX *vs.* CT | Very low | Study limitation; imprecision |
| ME *vs.* HT | Moderate | Imprecision |
| CT, combination therapy; ENS, electrical nerve stimulation; ESWT, extracorporeal shock wave therapy; EX, exercise; FIR, far-infrared ray; HT, heat; KT, Kinesio taping; ME, medication; US, ultrasound. | | |

**Supplementary material 9.2.** GRADE assessment for pairwise comparisons within the pressure pain threshold network

| Comparison | Confidence | Downgrading due to |
| --- | --- | --- |
| Manual *vs.* Control | Low | Study limitation; inconsistency |
| Laser *vs.* Control | Low | Study limitation; inconsistency |
| ENS *vs.* Control | Low | Study limitation; imprecision |
| ESWT *vs.* Control | Low | Study limitation; inconsistency |
| US *vs.* Control | Low | Study limitation; inconsistency |
| ME *vs.* Control | Low | Study limitation; imprecision |
| KT *vs.* Control | Low | Study limitation; imprecision |
| HT *vs.* Control | Moderate | Imprecision |
| FIR *vs.* Control | Low | Study limitation; imprecision |
| Manual *vs.* Laser | Low | Study limitation; imprecision |
| Manual *vs.* ESWT | Low | Study limitation; imprecision |
| Manual *vs.* US | Low | Study limitation; imprecision |
| Manual *vs.* KT | Low | Study limitation; imprecision |
| Manual *vs.* CT | Low | Study limitation; imprecision |
| Laser *vs.* ENS | Low | Study limitation; imprecision |
| ENS *vs.* ME | Low | Study limitation; imprecision |
| ENS *vs.* KT | Low | Study limitation; imprecision |
| ENS *vs.* HT | Moderate | Imprecision |
| ESWT *vs.* US | Low | Study limitation; imprecision |
| ESWT *vs.* EX | Low | Study limitation; imprecision |
| ESWT *vs.* FIR | Low | Study limitation; inconsistency |
| ESWT *vs.* CT | Low | Study limitation; imprecision |
| EX *vs.* CT | Low | Study limitation; imprecision |
| ME *vs.* HT | Moderate | Imprecision |
| CT, combination therapy; ENS, electrical nerve stimulation; ESWT, extracorporeal shock wave therapy; EX, exercise; FIR, far-infrared ray; HT, heat; KT, Kinesio taping; ME, medication; US, ultrasound. | | |

**Supplementary material 9.3.** GRADE assessment for pairwise comparisons within the pain-related disability network

| Comparison | Confidence | Downgrading due to |
| --- | --- | --- |
| Manual *vs.* Control | Low | Study limitation; inconsistency |
| Laser *vs.* Control | Very low | Study limitation; inconsistency^*^ |
| ENS *vs.* Control | Moderate | Imprecision |
| ESWT *vs.* Control | Very low | Study limitation; inconsistency^*^ |
| US *vs.* Control | Low | Study limitation; imprecision |
| ME *vs.* Control | Low | Study limitation; imprecision |
| KT *vs.* Control | Low | Study limitation; imprecision |
| HT *vs.* Control | Moderate | Imprecision |
| Manual *vs.* Laser | Low | Study limitation; imprecision |
| Manual *vs.* ESWT | Low | Study limitation; imprecision |
| Manual *vs.* US | Low | Study limitation; imprecision |
| Manual *vs.* CT | Low | Study limitation; imprecision |
| Manual *vs.* HT | Moderate | Inconsistency |
| Laser *vs.* ENS | Low | Study limitation; imprecision |
| Laser *vs.* ESWT | Very low | Study limitation; imprecision; inconsistency |
| Laser *vs.* CT | Moderate | Inconsistency |
| ENS *vs.* ME | Moderate | Imprecision |
| ENS *vs.* KT | Moderate | Imprecision |
| ENS *vs.* HT | Moderate | Imprecision |
| ESWT *vs.* US | Low | Study limitation; imprecision |
| ESWT *vs* EX | Low | Study limitation; imprecision |
| ESWT *vs.* CT | Low | Study limitation; imprecision |
| ESWT *vs.* HT | Low | Study limitation; inconsistency |
| EX *vs.* CT | Low | Study limitation; imprecision |
| US *vs.* CT | Low | Study limitation; imprecision |
| ME *vs.* HT | Moderate | Imprecision |
| ^*^Inconsistency, 2 downgrades.  CT, combination therapy; ENS, electrical nerve stimulation; ESWT, extracorporeal shock wave therapy; EX, exercise; HT, heat; KT, Kinesio taping; ME, medication; US, ultrasound. | | |

**Supplementary material 10.1.** Funnel plot for pain intensity. CN, control; CT, combination therapy; ENS, electrical nerve stimulation; ESWT, extracorporeal shock wave therapy; EX, exercise; FIR, far-infrared ray; HT, heat; KT, Kinesio taping; LT, laser therapy; ME, medication; MT, manual therapy; US, ultrasound.


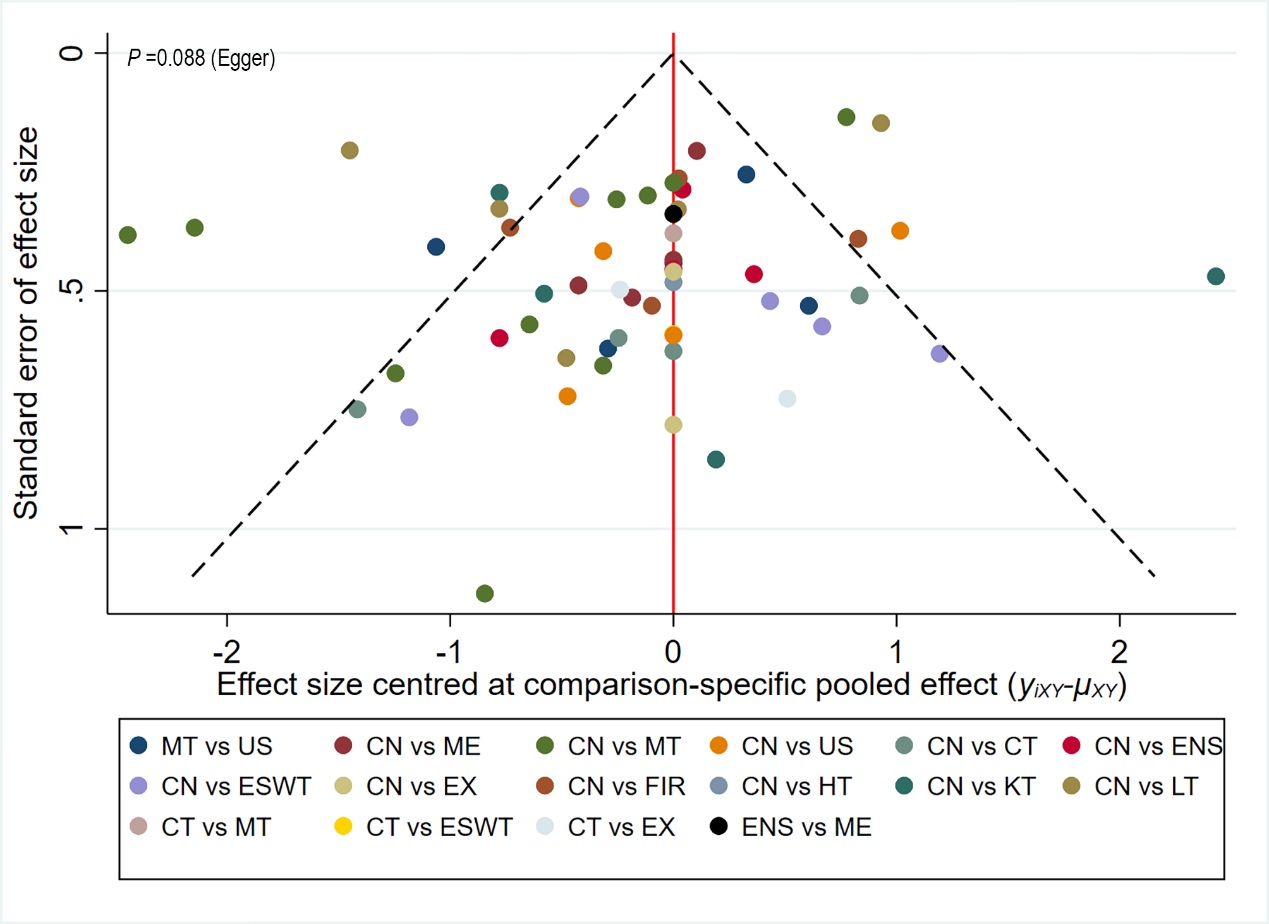


**Supplementary material 10.2.** Funnel plot for pressure pain threshold. CN, control; CT, combination therapy; ENS, electrical nerve stimulation; ESWT, extracorporeal shock wave therapy; EX, exercise; FIR, far-infrared ray; HT, heat; KT, Kinesio taping; LT, laser therapy; ME, medication; MT, manual therapy; US, ultrasound.


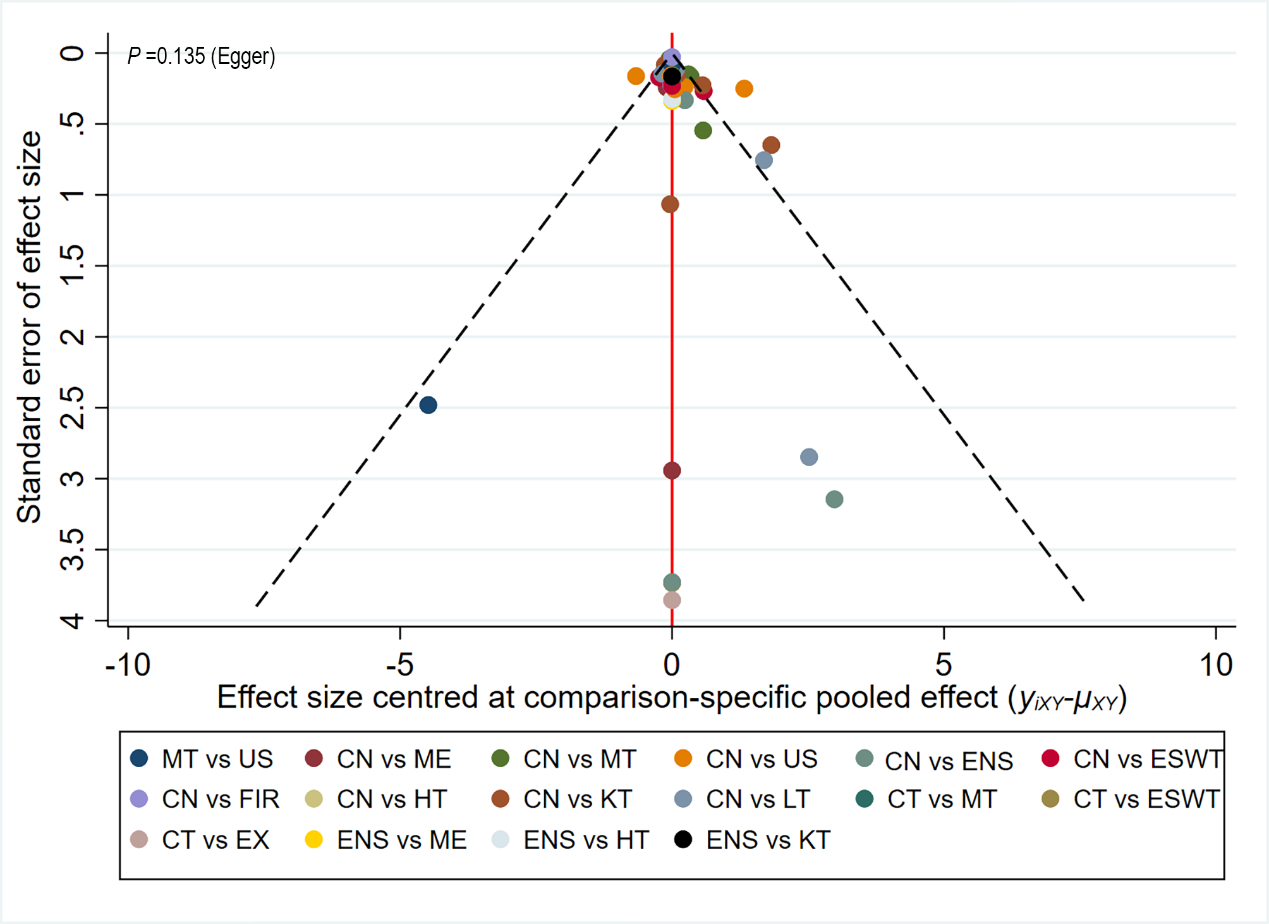


**Supplementary material 10. 3.** Funnel plot for pain-related disability. CN, control; CT, combination therapy; ENS, electrical nerve stimulation; ESWT, extracorporeal shock wave therapy; EX, exercise; HT, heat; KT, Kinesio taping; LT, laser therapy; ME, medication; MT, manual therapy; US, ultrasound.


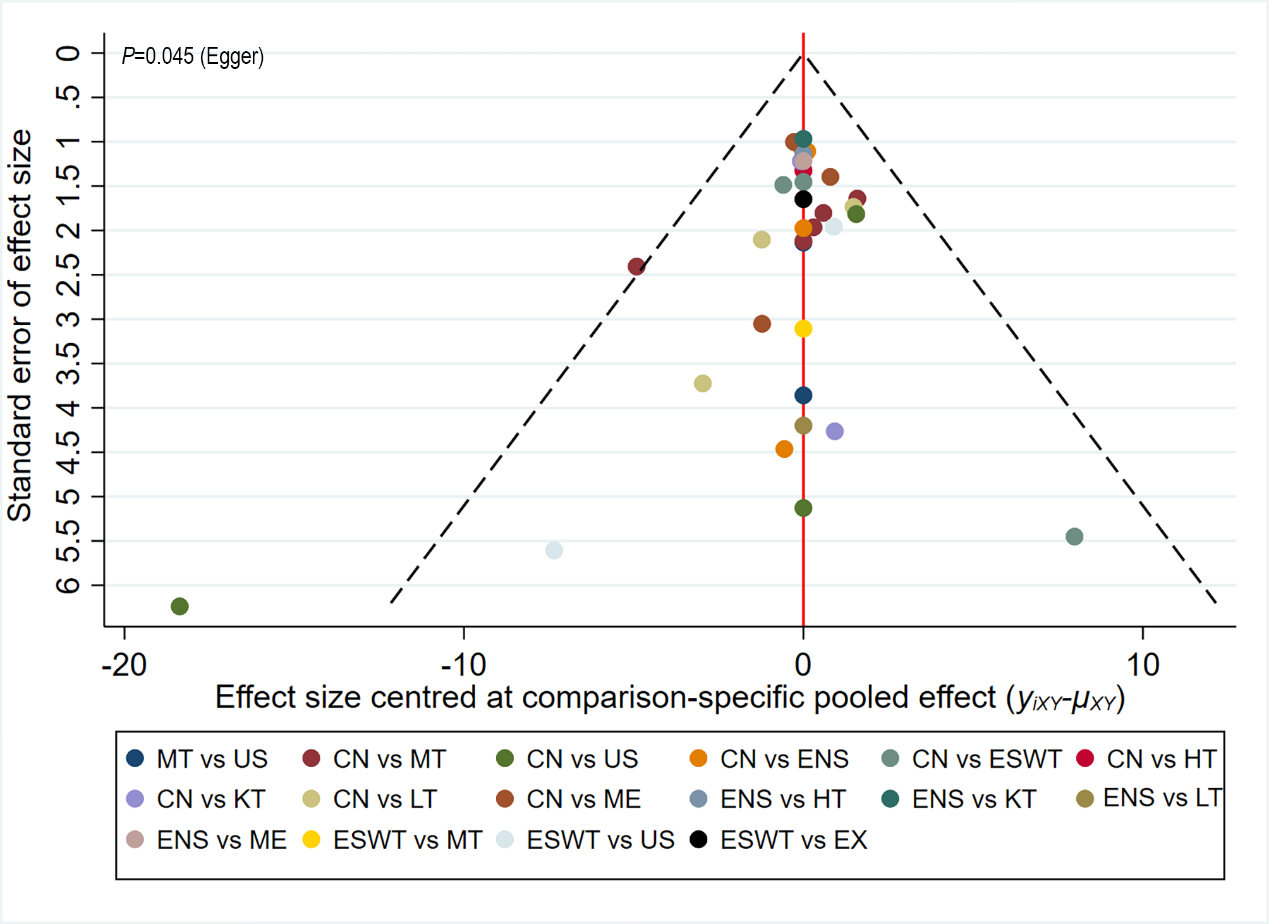


**Supplementary material 11**

| Summary of changes between original protocol and final manuscript | | | |
| --- | --- | --- | --- |
| Changes | Original protocol | Final manuscript | Reasons |
| Electronic Databases | PubMed, Ovid JBI, CINAHL Complete, Web of Science, Cochrane, Scopus, and China National Knowledge Infrastructure (CNKI) and Wanfang database. | PubMed, Embase, CINAHL Complete, Web of Science, Cochrane, and Scopus. | The database of Embase was more comprehensive and professional than Ovid JBI  and Chinese database did not contain the relevant literature. |
| Retrieval years | 2012 to 2023 | Inception through 2023 | In order to search more articles and to make the results more credible. |
| Outcomes | Pain intensity, pressure pain threshold, pain-related disability, and quality of life. | Pain intensity, pressure pain threshold, pain- related disability. | Articles (only 8) of RCTs with quality of life were insufficient to conduct network meta-analysis. |
